# Supplementary material for: Geospatial Analyses of Recent Household Surveys to Assess Changes in the Distribution of Zero-Dose Children and Their Associated Factors before and during the COVID-19 Pandemic in Nigeria
Source: Vaccines (Basel). 2023 Dec 8;11(12):1830. doi: 10.3390/vaccines11121830 (PMC10747017; doi:10.3390/vaccines11121830)
Supplement: Supplementary file 1 [file vaccines-11-01830-s001.zip › vaccines-2711642-supplementary.pdf]

Geospatial analyses of recent household surveys to assess changes in the distribution of zero-dose children and associated factors before and during the Covid-19 pandemic in Nigeria

Justice MK Aheto, Iyanuloluwa Olowe, Ho Man Theophilus Chan, Adachi Ekeh, Boubacar Dieng, Biyi Fafunmi, Hamidreza Setayesh, Brian Atuhaire, Jessica Crawford, Andrew J Tatem, C. Edson Utazi

Supporting information

Table S1: Description and coding of outcome variables and covariate factors

Variable name and labels	Description and coding	Reference category
**Outcome variables (binomial)**		
Received DTP1 (PENTA1)	No/don't know - 0, reported by mother/ vaccination date on card/ vaccination marked on card - 1	No/don't know - 0
Received MCV1	No/don't know - 0, reported by mother/ vaccination date on card/ vaccination marked on card - 1	No/don't know - 0
Composite coverage (i.e., receipt of at least one of the 4 basic vaccines – BCG, OPV0, DTP1 and MCV1)	Yes – 1, No – 0 (BCG and OPV0 coded similar to DTP1 and MCV1)	No - 0
**Predictor variables**		
Sex of child	Male – 1, female – 2	Male – 0
Skilled birth attendance	No – 0, yes - 1	No – 0
Birth quarter	Jan-Mar – 0, Apr-Jun – 1, Jul-Sep – 2, Oct-Dec - 3	Jan-March - 0
Mother received tetanus (TT)	No - 0, yes - 1	No - 0
ANC visits	0 – 0, 1-3 – 1, ≥4 - 2	0 - 0
Mother's age	15-19 – 0, 20-29 – 1, 30-39 – 2, 40-49 – 3	15-19 – 0
Marital status of mother	Never in union - 0, married/ living with partner – 1, divorced/widowed/ no longer living together/separated – 2	Never in union – 0
Mother's education	No education – 0, primary – 1, junior/secondary – 2, higher/tertiary - 3	No education – 0
Mother's religion	Islam – 0, Christian/Traditionalist/others – 1	Islam – 0

Access to media	No - 0, yes (listens to radio or tv or reads newspaper at least once in a week) - 1	No - 0
Mother's access to mobile phone/internet	No - 0, yes - 1	No - 0
Mother's land ownership	No - 0, yes - 1	No - 0
Mother had health insurance	No - 0, yes - 1	No - 0
Mother's ethnicity	Hausa/Fulani - 0, Yoruba - 1, Igbo - 2, Others (Kanuri, Tiv, etc.) - 3	Hausa/Fulani - 0
Sex of household head	Female - 0, male - 1	Female - 0
Household wealth	Poorer/poorest - 0, middle - 1, rich/richest - 2	Poorer/poorest - 0
Access to bank account	No - 0, yes - 1	No - 0
Household size	Large (>=9) - 0, medium (5 to 8) - 1, small (<=4) - 2	Large (>=9) - 0
Length of stay in household	<1year/visitor - 0, 1-3years - 1, 4-5years - 2, >5years/always - 3	<1year/visitor - 0
Rural/urban	Rural - 0, urban - 1	Rural - 0
Region	North west - 0, north east - 1, north central - 2, south east - 3, south south - 4, south west - 5	North west - 0
Livestock density index	Lower (0-60.2) - 0, medium (60.3-123.7) - 1, higher (123.8-660.0) - 2	Higher (123.8-660.0) - 2
Travel time (motorized)	Lower (0-2.4) - 0, medium (2.5-8.3) - 1, higher (8.4-181.9) - 2	Higher (8.4-181.9) - 2
Distance to coastline	Lower (0.5-0.135.3) - 0, medium (135.4-472.7) - 1, higher (472.8-999.2) - 2	Higher (472.8-999.2) - 2
Distance to edge of cultivation area	Lower (-12.9- -0.5) - 0, medium (-0.4-0.3) - 1, higher (0.31-14.0) - 2	Higher (0.31-14.0) - 2
Distance to conflict area	Lower (11.4-142.2) - 0, medium (142.3-347.2) - 1, higher (347.3-10790.2) - 2	Higher (347.3-10790.2) - 2
Number of wet days	Lower (37.4-82.7) - 0, medium (82.8-123.0) - 1, higher (123.1-179.8) - 2	Higher (123.1-179.8) - 2
Proximity to national borders	Lower (0.6-69.5) - 0, medium (69.6-158.0) - 1, higher (158.1-394.0) - 2	Higher (158.1-394.0) - 2
Proximity to protected areas	Lower (0.3-132.0) - 0, medium (132.1-272.2) - 1, higher (272.3-699.5) - 2	Higher (272.3-699.5) - 2
Day time land surface temperature	Lower (25.9-31.1) - 0, medium (31.2-34.9) - 1, higher (35.0-38.7) - 2	Higher (35.0-38.7) - 2
Enhanced vegetation index	Lower (375.9-2340.8) - 0, medium (2340.9-3067.1) - 1, higher (3067.2-4322.4) - 2	Higher (3067.2-4322.4) - 2

Poverty index	Lower (0.06-0.72) - 0, medium (0.73-0.82) - 1, higher (0.83-0.94) - 2	Higher (0.83-0.94) - 2
Slope	Lower (0.27-1.06) - 0, medium (1.07-2.04) - 1, higher (2.05-14.9) - 2	Higher (2.05-14.9) - 2

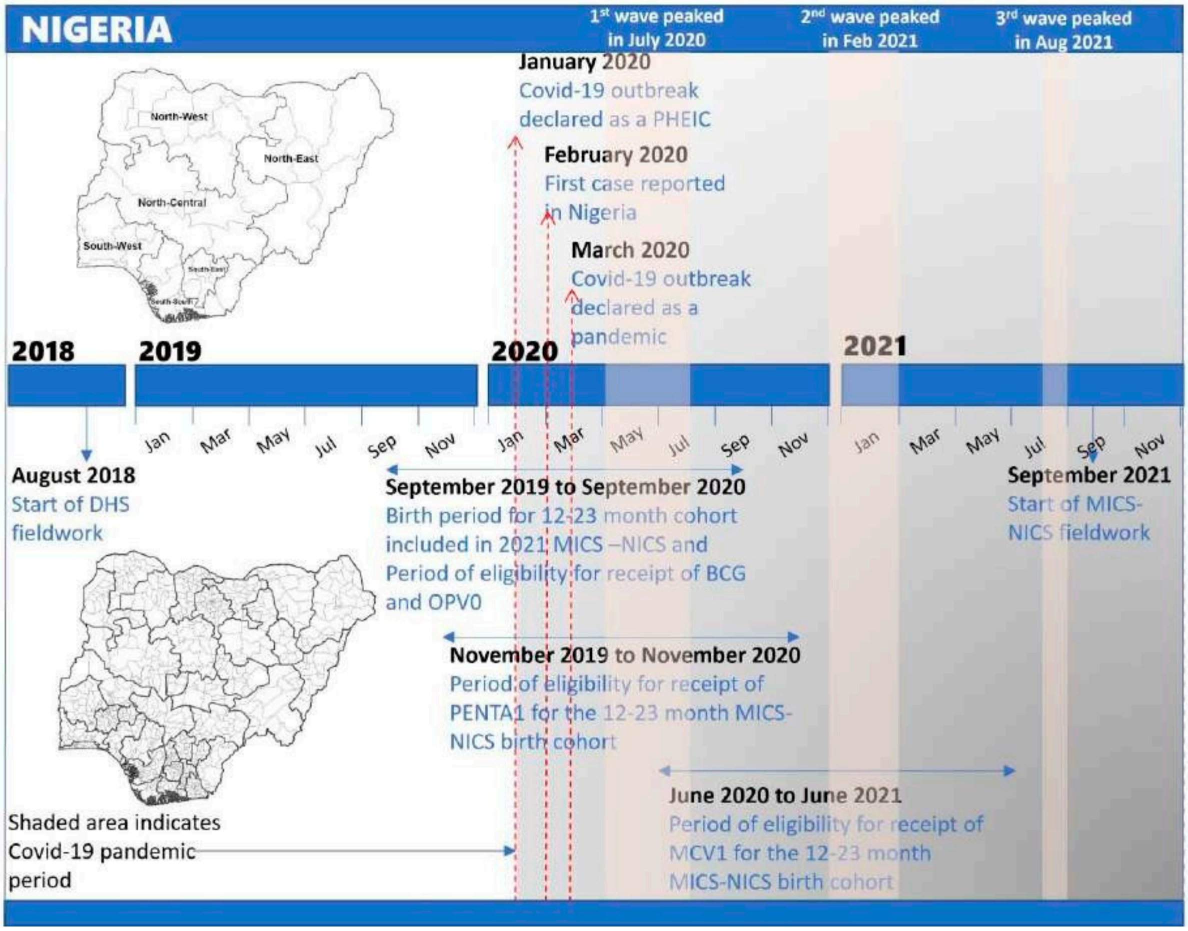

Figure S1: Timeline of the 2018 DHS and 2021 MICS-NICS surveys in Nigeria showing the time intervals the 12-23-month birth cohort analysed became eligible to receive the vaccines included in the study. The timeline also shows the history of the Covid-19 pandemic in Nigeria and important related events.

Geospatial covariate description and processing steps

The geospatial covariates used in the analyses, as described in the main manuscript were travel time to the nearest health facility providing routine immunization (RI) services, livestock density and Enhanced Vegetation Index (EVI). These covariates were obtained from the sources mentioned in Table S2 and were processed as follows. Using ESRI ArcGIS v10.7, standardised gridded covariate layers were processed at 1km x 1 km for Nigeria from the raw data sets. For EVI, the data for the raster layers were taken from the 5-year period prior to the DHS survey, however, this was not possible for the other covariates. These layers were further processed to obtain the average EVI over the period. The livestock density index used in our work was obtained as the average of the individual livestock densities (i.e., cattle, chicken, goat, pig, and sheep) listed in Table S2. Following these, covariate data values were extracted from each raster layer for each DHS cluster location using R. Prior to extraction, considerations for urban and rural clusters were made as in previous work [1] and buffers were created for data extraction to calculate a mean value at 2 km for urban areas and 5 km for rural areas. We present the surfaces of the resulting geospatial covariates and the extracted values at the cluster level in Figure S1.

Table S2: Description and sources of geospatial covariates used in our analyses.

1	Cattle density	No. of cattle per sq km	2010	Continuous	Gilbert, M. *et al.* (2018) Global Distribution Data for Cattle, Buffaloes, Horses, Sheep, Goats, Pigs, Chickens and Ducks in 2010. Nature Scientific data, 5:180227. doi: 10.1038/sdata.2018.227
2	Chicken density	No. of chickens per sq km	2010	Continuous	Gilbert, M. *et al.* (2018) Global Distribution Data for Cattle, Buffaloes, Horses, Sheep, Goats, Pigs, Chickens and Ducks in 2010. Nature Scientific data, 5:180227. doi: 10.1038/sdata.2018.227
3	Goat density	No. of goats per sq km	2010	Continuous	Gilbert, M. *et al.* (2018) Global Distribution Data for Cattle, Buffaloes, Horses, Sheep, Goats, Pigs, Chickens and Ducks in 2010. Nature Scientific data, 5:180227. doi: 10.1038/sdata.2018.227
4	Pig density	No. Of pigs per sq km	2010	Continuous	Gilbert, M. *et al.* (2018) Global Distribution Data for Cattle, Buffaloes, Horses, Sheep, Goats, Pigs, Chickens and Ducks in 2010. Nature Scientific data, 5:180227. doi: 10.1038/sdata.2018.227
5	Sheep density	No. Of sheep per sq km	2010	Continuous	Gilbert, M. *et al.* (2018) Global Distribution Data for Cattle, Buffaloes, Horses, Sheep, Goats, Pigs, Chickens and Ducks in 2010. Nature Scientific data, 5:180227. doi: 10.1038/sdata.2018.227
6	Travel time (motorized)	Minutes	2018	Continuous	[*Produced from locations of health facilities in Nigeria using the methodology in*] Weiss, D.J. *et al*. (2018). A global map of travel time to cities to access inequalities in accessibility in 2015. *Nature*.
7	Poverty index				Tatem AJ, Gething PW, Bhatt S, Weiss D and Pezzulo C (2013) Pilot high resolution poverty maps, University of Southampton/Oxford. (http://www.worldpop.org.uk/resources/docs/WorldPop-poverty-mapping-methods.pdf)<br>G-Econ Project, Yale University, 2009. (http://gecon.yale.edu/)
8	Average Modis Enhanced Vegetation Index between 2013 and 2018	EVI (0 to 1)	2013-2018	Continuous	Didan, K. (2015). MOD13A3 MODIS/Terra vegetation Indices Monthly L3 Global 1km SIN Grid V006. NASA EOSDIS LP DAAC.
9	Day time land surface temperature	Kelvin, scaled 0.02		Continuous	Wan, Z., Hook, S., Hulley, G. (2021). MODIS/Terra Land Surface Temperature/Emissivity 8-Day L3 Global 1km SIN Grid V061 [Data set]. NASA EOSDIS Land Processes DAAC. https://doi.org/10.5067/MODIS/MOD11A2.061
10	Slope	Degrees	2000	Continuous	WorldPop (www.worldpop.org - School of Geography and Environmental Science, University of Southampton; Department of Geography and Geosciences, University of Louisville; Departement de Geographie, Universite de Namur) and Center for International Earth Science Information Network (CIESIN), Columbia University (2018). Global High Resolution Population Denominators Project - Funded by The Bill and Melinda Gates Foundation (OPP1134076). https://dx.doi.org/10.5258/SOTON/WP00644

11	Proximity to protected areas	km		Continuous	WorldPop (www.worldpop.org - School of Geography and Environmental Science, University of Southampton; Department of Geography and Geosciences, University of Louisville; Departement de Geographie, Universite de Namur) and Center for International Earth Science Information Network (CIESIN), Columbia University (2018). Global High Resolution Population Denominators Project - Funded by The Bill and Melinda Gates Foundation (OPP1134076). https://dx.doi.org/10.5258/SOTON/WP00644
12	Proximity to national borders	km		Continuous	Department of State's Office of the Geographer. 2018. "Department of State Large-Scale International Boundary (LSIB)." Via ESRI.<br>https://www.arcgis.com/home/item.html?id=3e650cfe52b84bffacb86d028f1f0514#

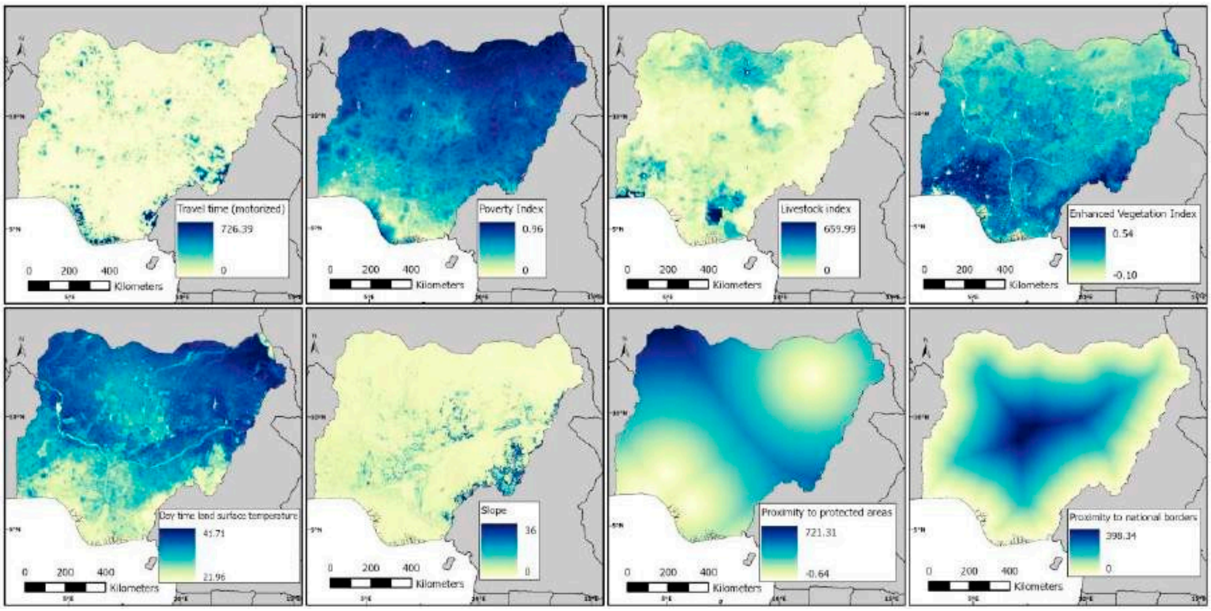

Figure S2: Geospatial covariates used in our analyses (top row) and the corresponding cluster-level values (bottom row).

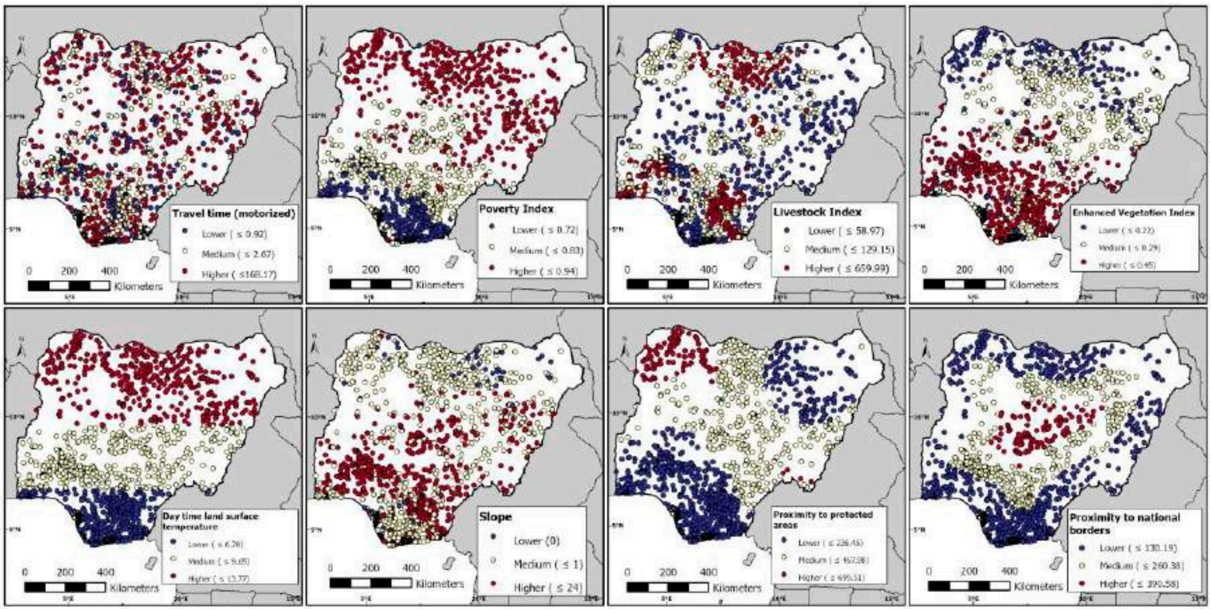

Figure S3. Classification of the geospatial covariates included in the multi-level analyses using the tertiles of the distribution of each covariate.

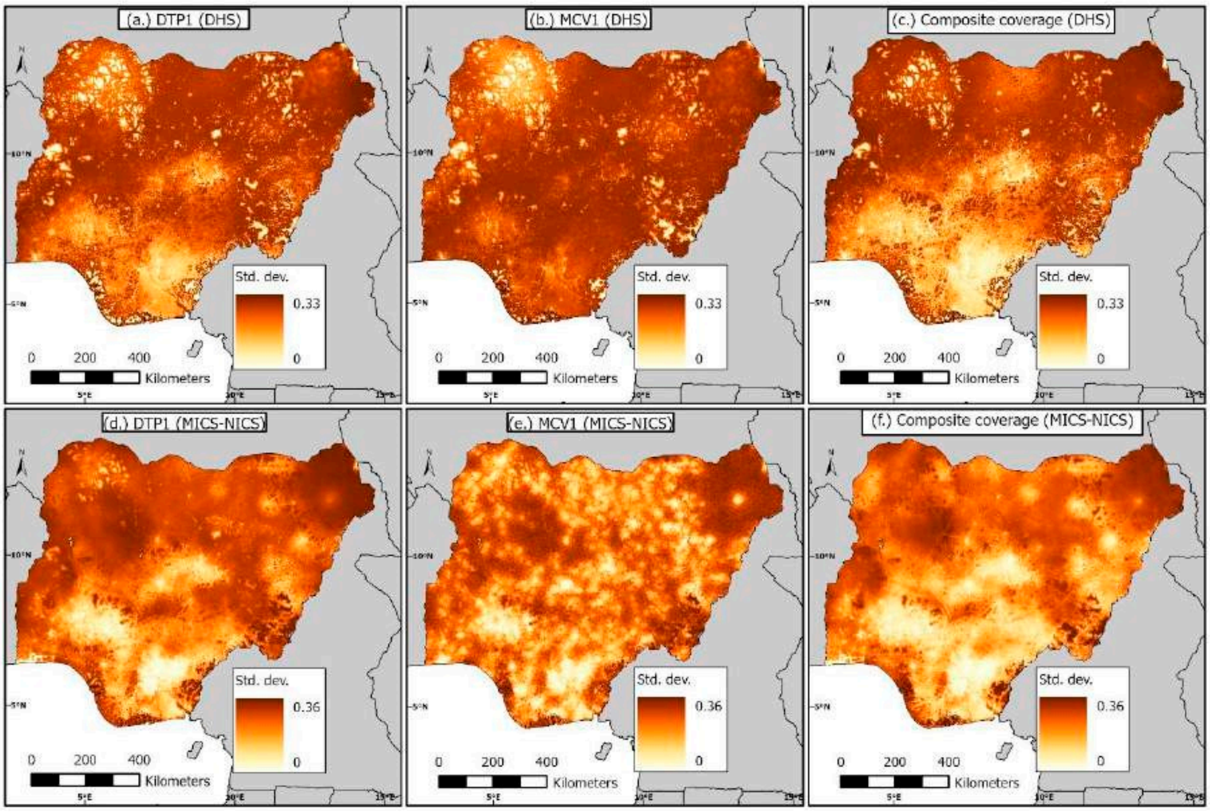

Figure S4: Uncertainty estimates associated with the coverage estimates for DTP1 (a), MCV1 (b), and composite coverage (c) for 2018 DHS (top panel) and 2021 MICS-NICS (bottom panel) for DTP1 (d), MCV1 (e) and composite coverage (f) at 1 x 1km resolution for Nigeria.

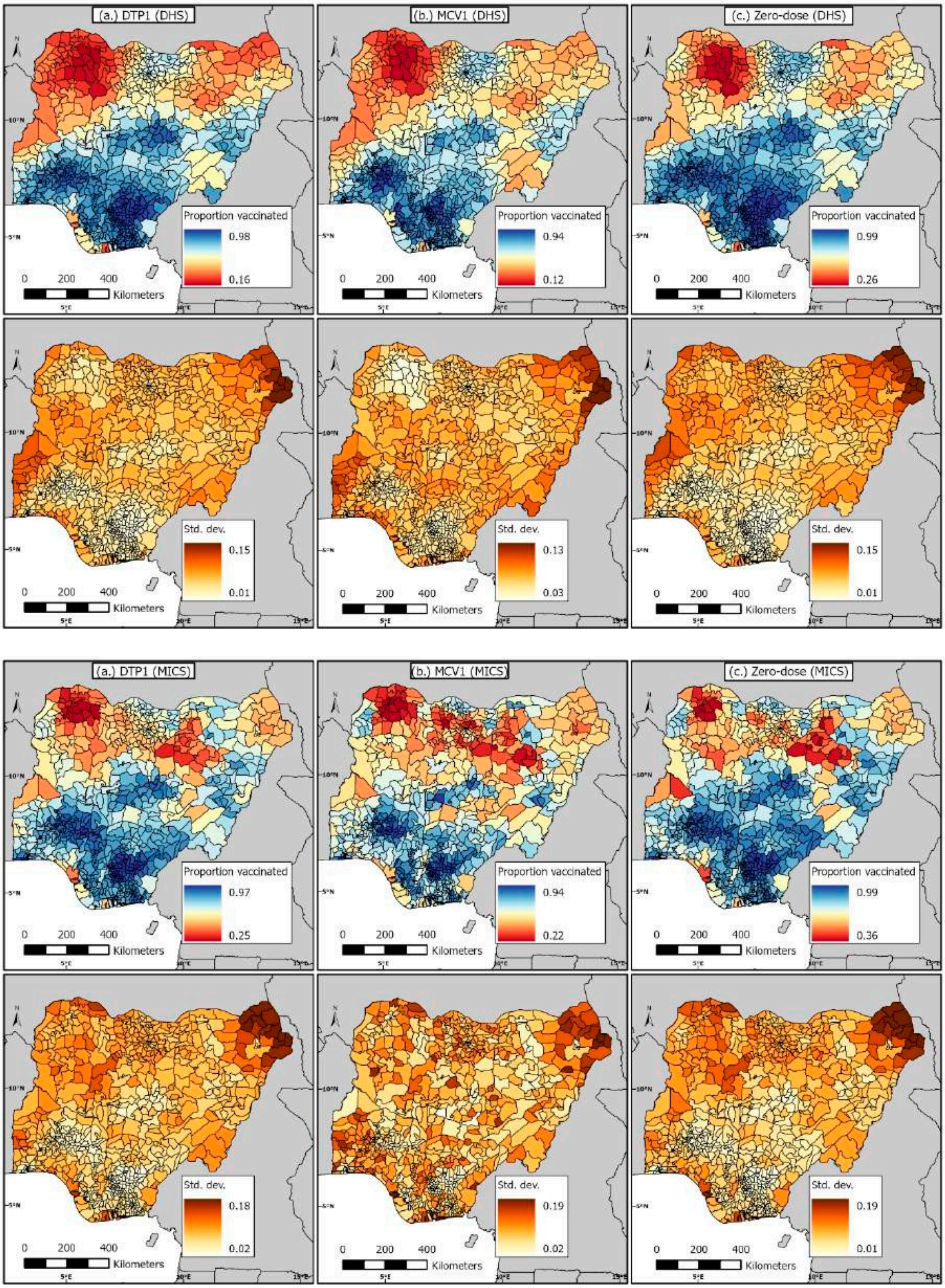

Figure S5: District level coverage estimates and associated uncertainties for DTP1, MCV1, and Composite Coverage (CC) for both DHS and MICS-NICS.

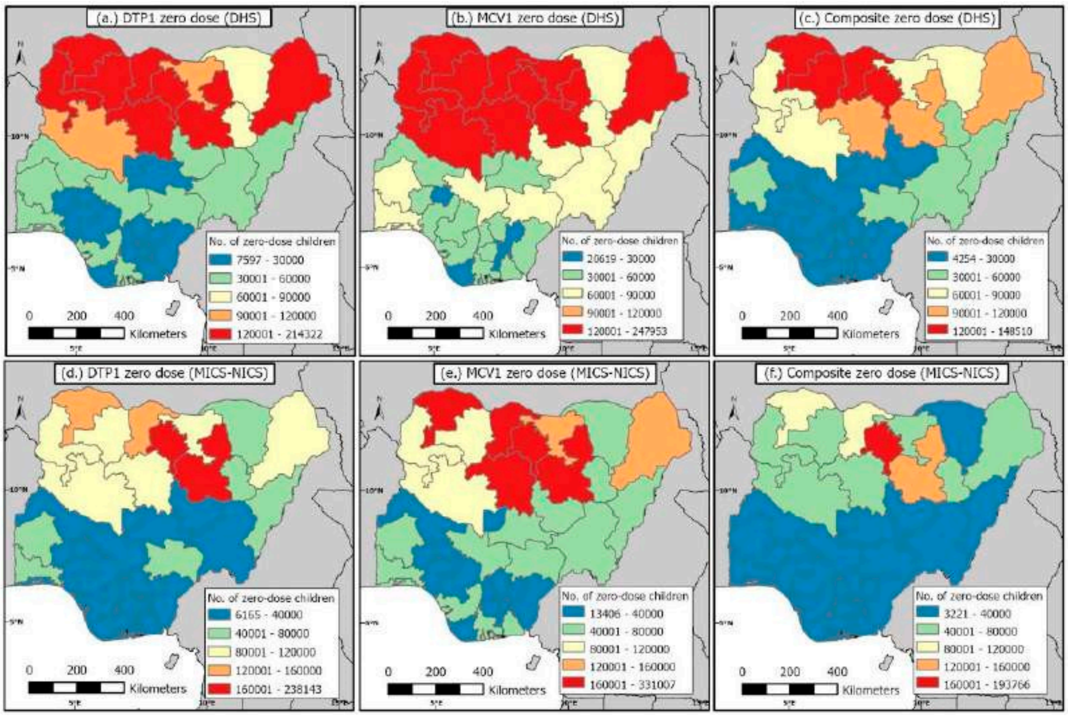

Figure S6: Estimates of numbers of DTP, MCV and composite zero-dose children at the state level before the pandemic in 2018 (DHS) and during the pandemic in 2021 (MICS-NICS)

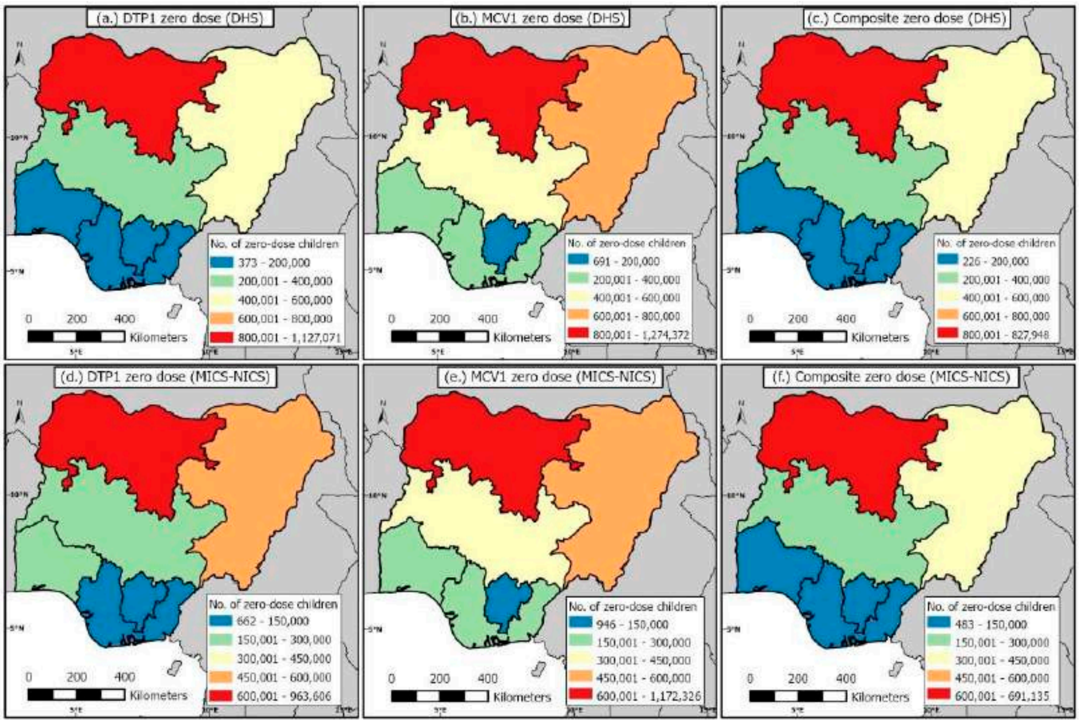

Figure S7: Estimates of numbers of DTP, MCV and composite zero-dose children at the regional level before the pandemic in 2018 (DHS) and during the pandemic in 2021 (MICS-NICS)

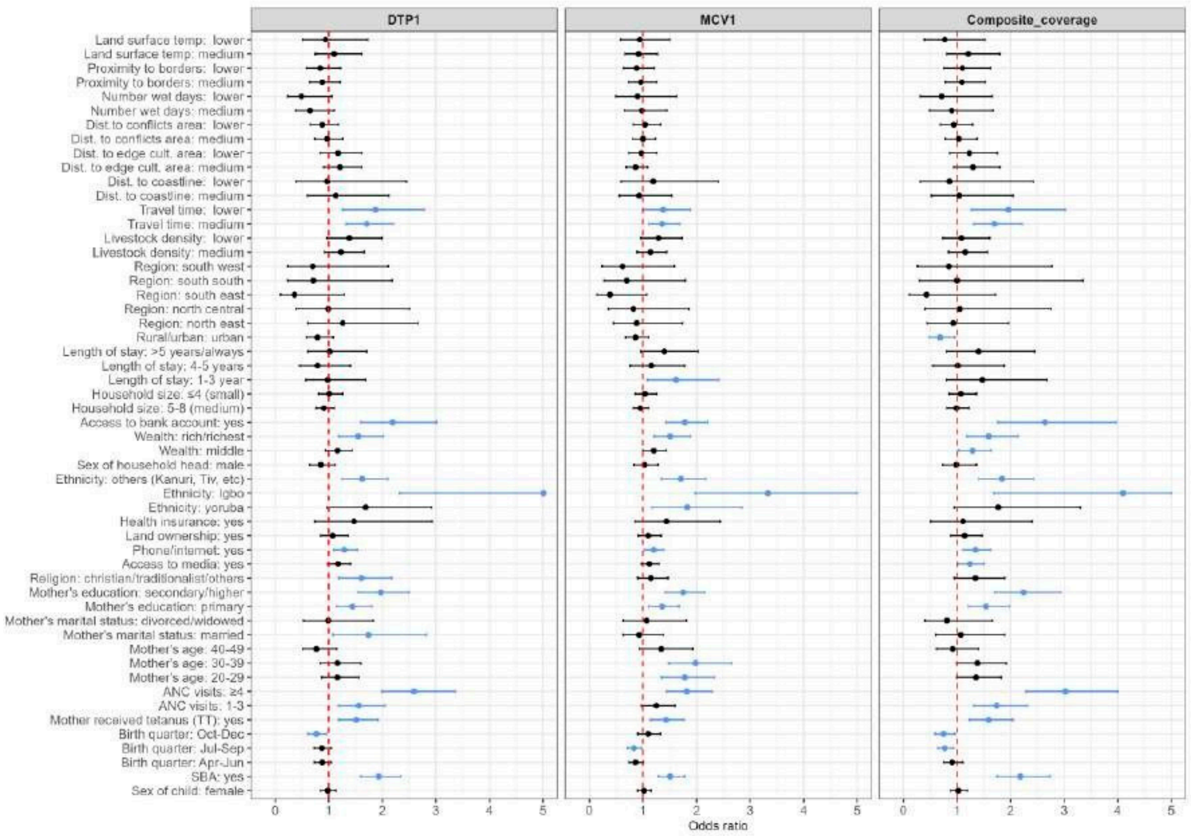

Figure S8: Risk factors associated with DTP1, MCV1, and composite coverage before the pandemic in 2018 (DHS) at the national level. The odds ratios and 95% credible intervals for significant risk factors are coloured in blue. Also, the upper limit for Igbo ethnicity is 10.85 for DTP1, 5.6 for MCV1, and 9.94 for composite coverage.

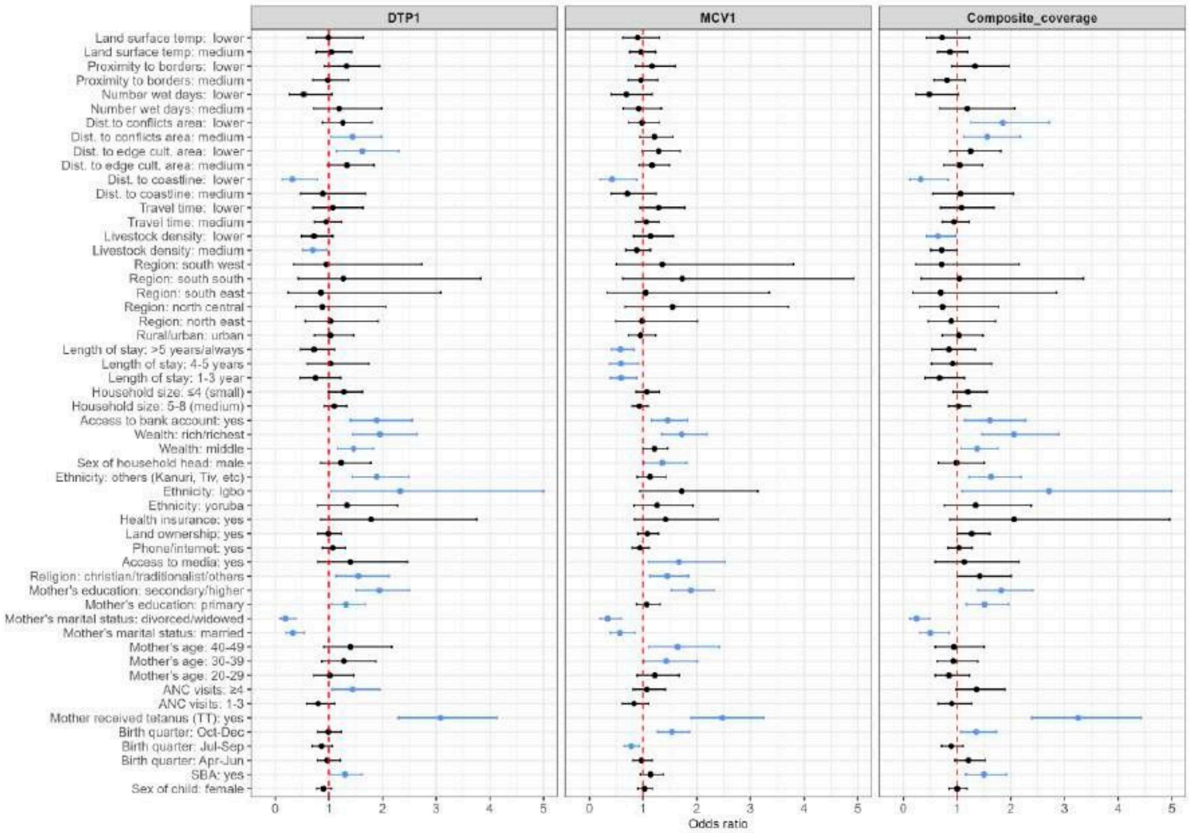

Figure S9: Risk factors associated with DTP1, MCV1, and composite coverage during the pandemic in 2021 (MICS-NICS) at the national level. The odds ratios and corresponding 95% credible intervals for significant risk factors are coloured in blue. Also, the upper limit for Igbo ethnicity is 5.21 for DTP1, and 6.77 for composite coverage.

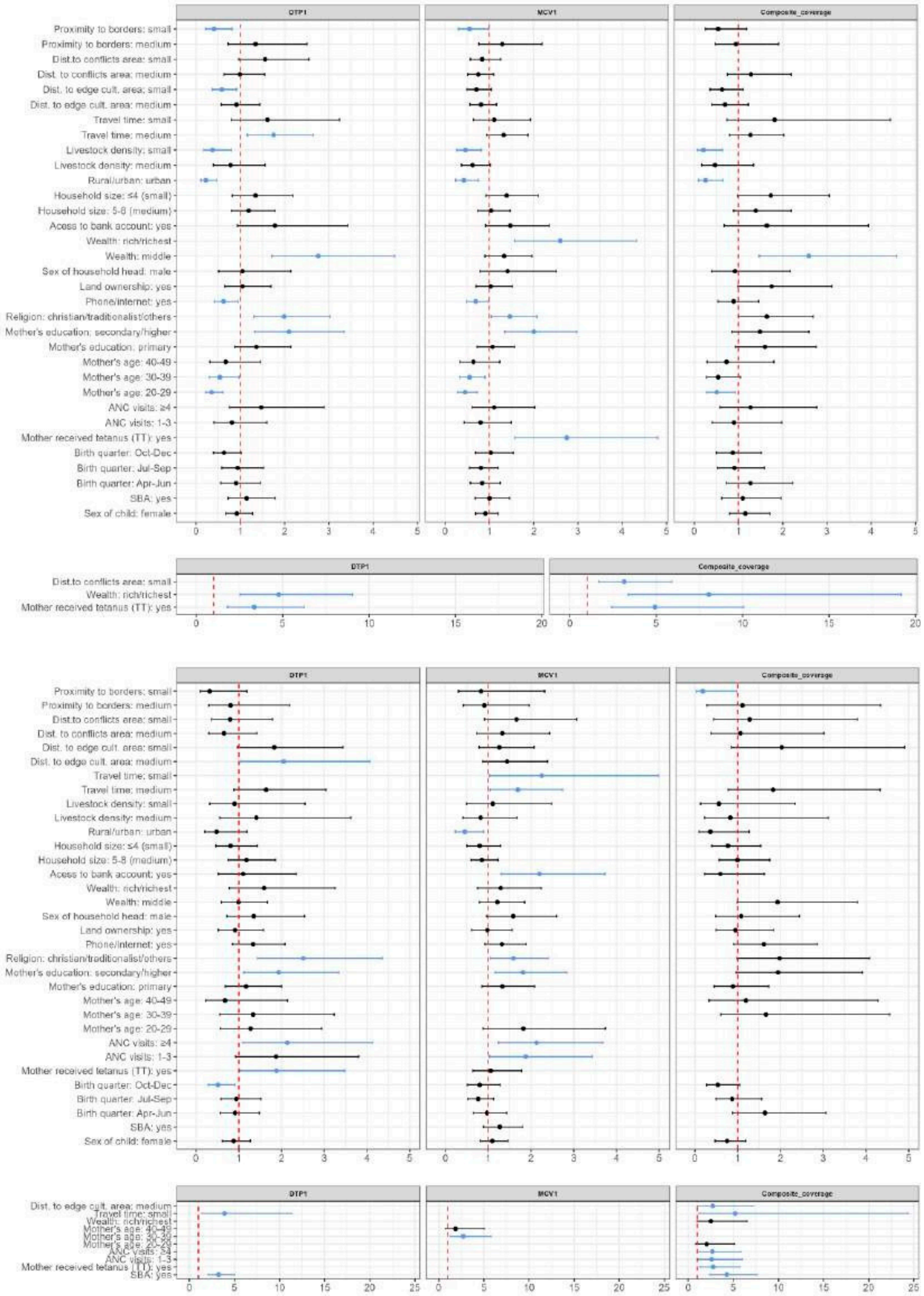

Figure S10: Risk factors associated with DTP1, MCV1, and composite coverage before and during the pandemic in 2018 (DHS - top panel) and 2021 (MICS-NICS - bottom panel) in the north central region. The odds ratios and corresponding 95% credible intervals for significant risk factors are coloured in blue.

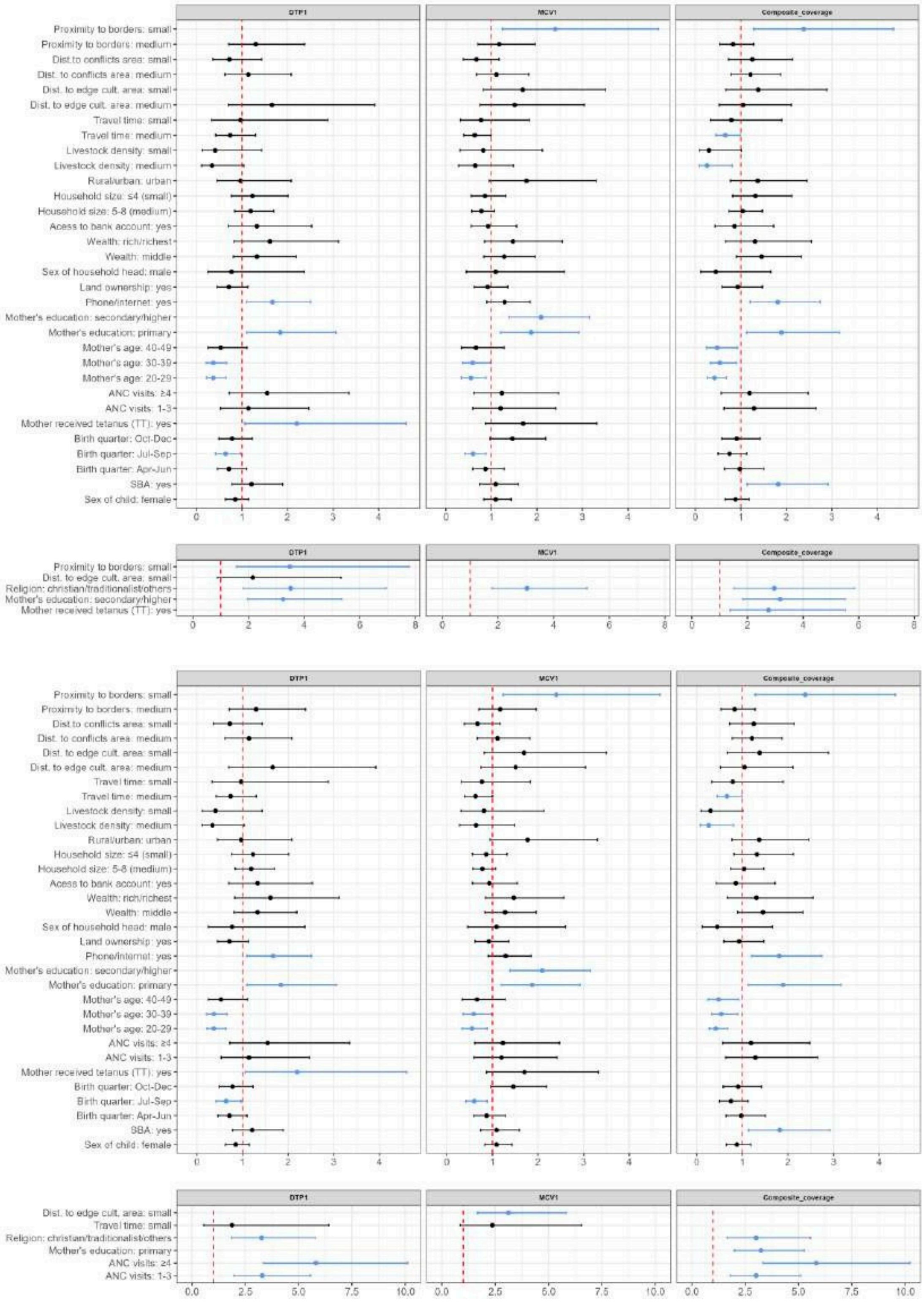

Figure S11: Risk factors associated with DTP1, MCV1, and composite coverage before and during the pandemic in 2018 (DHS - top panel) and 2021 (MICS-NICS - bottom panel) in the northeast region. The odds ratios and corresponding 95% credible intervals for significant risk factors are coloured in blue.

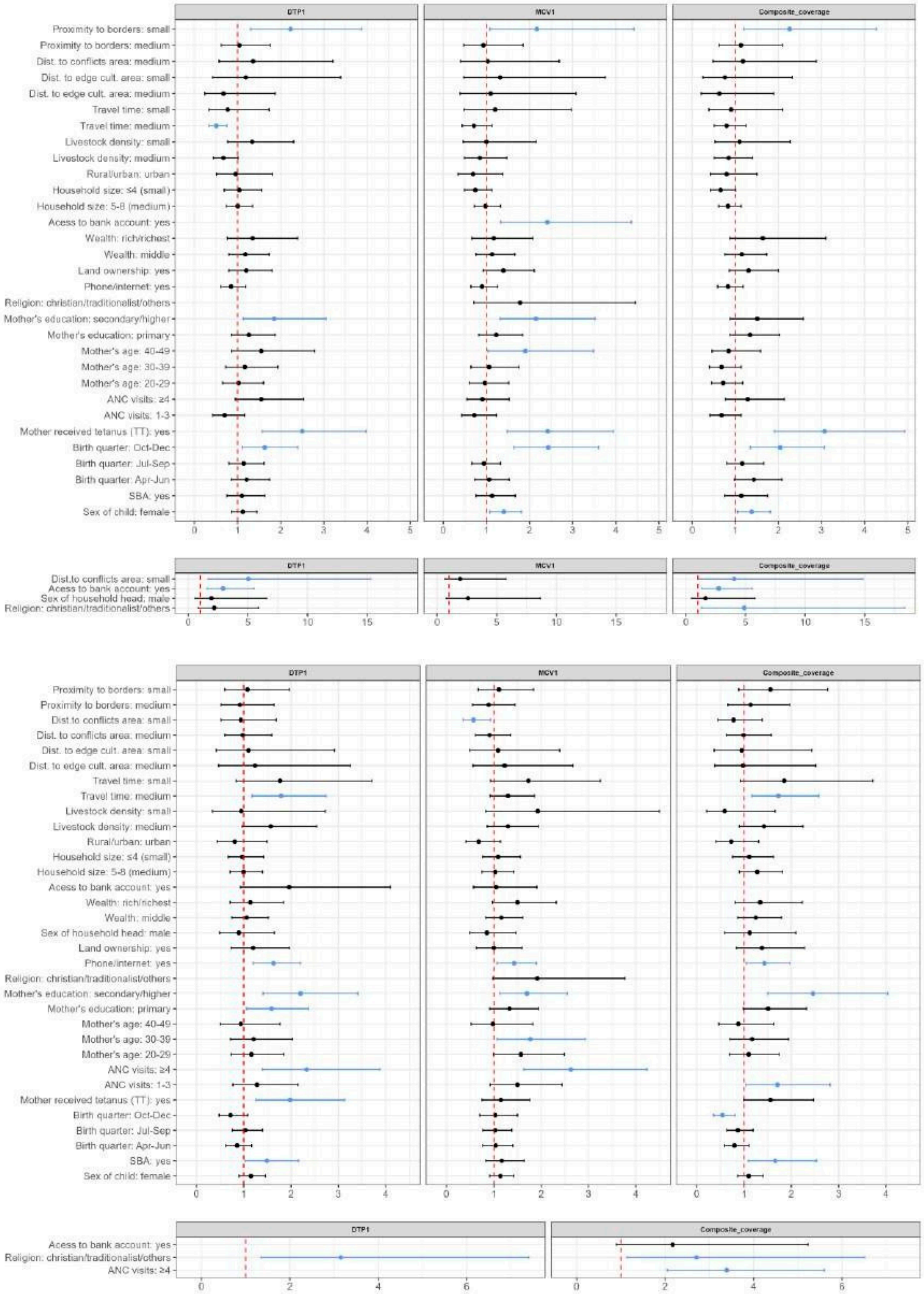

Figure S12: Risk factors associated with DTP1, MCV1, and composite coverage before and during the pandemic in 2018 (DHS - top panel) and 2021 (MICS-NICS - bottom panel) in the northwest region. The odds ratios and corresponding 95% credible intervals for significant risk factors are coloured in blue.

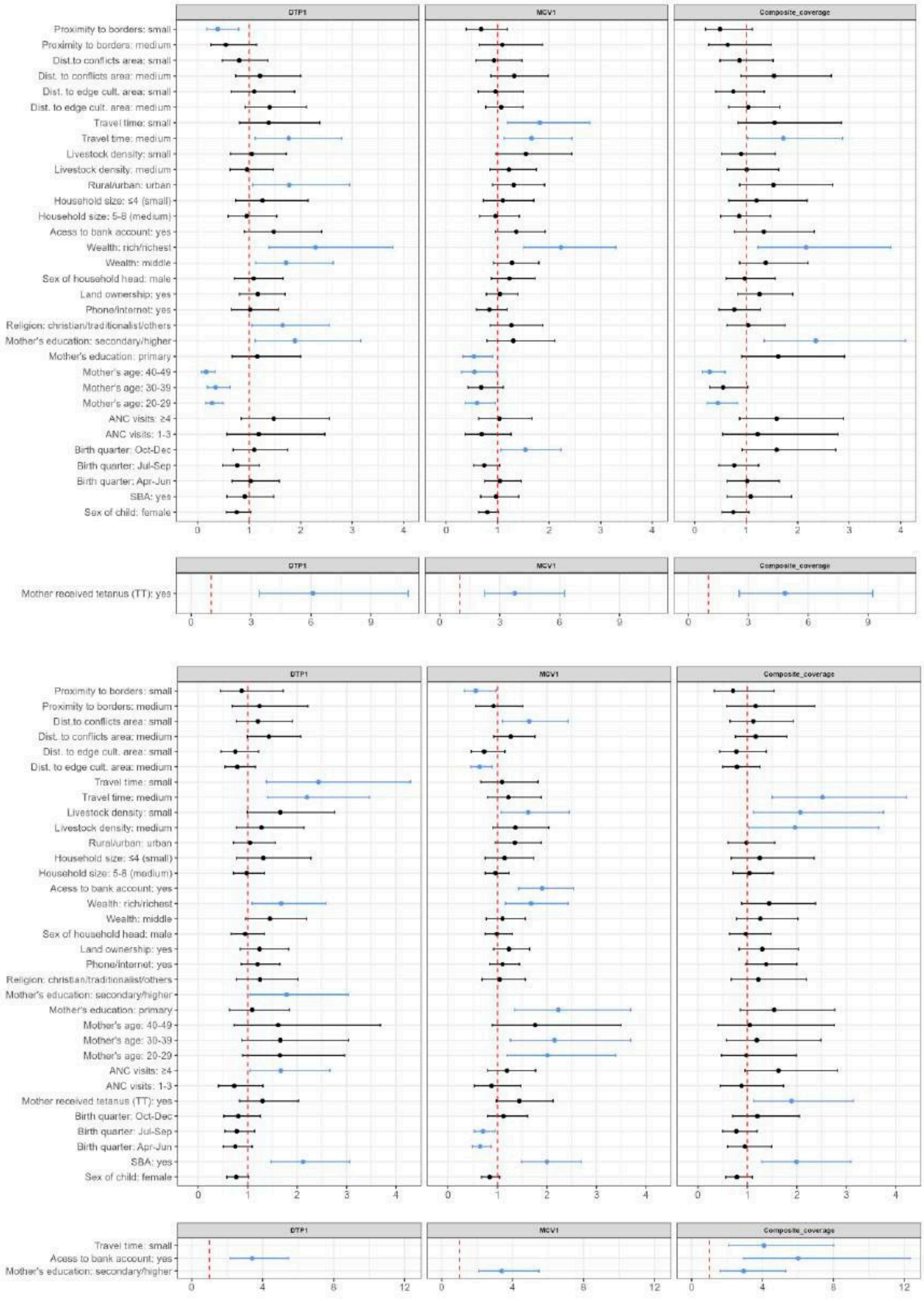

Figure S13: Risk factors associated with DTP1, MCV1, and composite coverage before and during the pandemic in 2018 (DHS - top panel) and 2021 (MICS-NICS - bottom panel) in the southern region. The odds ratios and corresponding 95% credible intervals for significant risk factors are coloured in blue.

Table S3: Distribution of sample characteristics of the study population by receipt of vaccine status for MICS-NICS 2021 and DHS 2018

	MICS 2021						DHS 2018					
	Received DTP1		Received MCV1		Receipt of Zero dose		Received DTP1		Received MCV1		Receipt of Zero dose	
**Characteristics**	**No (%)**	**Yes (%)**	**No (%)**	**Yes (%)**	**No (%)**	**Yes (%)**	**No (%)**	**Yes (%)**	**No (%)**	**Yes (%)**	**No (%)**	**Yes (%)**
**Total**	1,581 (29.0)	3,878 (71.0)	2,125 (38.9)	3,334 (61.1)	4,334 (79.4)	1,125 (20.6)	2,252 (35.2)	4,141 (64.8)	2,982 (46.6)	3,411 (53.4)	4,715 (73.8)	1,678 (26.2)
**Sex of child**												
Male	794 (28.2)	2,024 (71.8)	1,096 (38.9)	1,722 (61.1)	2,243 (79.6)	575 (20.4)	1,164 (35.1)	2,151 (64.9)	1,547 (46.7)	1,768 (53.3)	2,435 (73.5)	880 (26.5)
Female	787 (29.8)	1,854 (70.2)	1,029 (39.0)	1,612 (61.0)	2,091 (79.2)	550 (20.8)	1,088 (35.3)	1,990 (64.7)	1,435 (46.6)	1,643 (53.4)	2,280 (74.1)	798 (25.9)
**Skilled birth attendance**												
No	1,085 (44.0)	1,382 (56.0)	1,282 (52.0)	1,185 (48.0)	1,664 (67.5)	803 (32.5)	1,858 (53.4)	1,619 (46.6)	2,162 (62.2)	1,315 (37.8)	2,029 (58.4)	1,448 (41.6)
Yes	300 (14.1)	1,832 (85.9)	560 (26.3)	1,572 (73.7)	1,959 (91.9)	173 (8.1)	394 (13.5)	2,522 (86.5)	820 (28.1)	2,096 (71.9)	2,686 (92.1)	230 (7.9)
**Birth quarter**												
January - March	373 (28.4)	941 (71.6)	514 (39.1)	800 (60.9)	1,036 (78.8)	278 (21.2)	660 (33.3)	1,323 (66.7)	876 (44.2)	1,107 (55.8)	1,506 (75.9)	477 (24.1)
April - June	407 (28.4)	1,027 (71.6)	564 (39.3)	870 (60.7)	1,153 (80.4)	281 (19.6)	636 (35.9)	1,138 (64.1)	840 (47.4)	934 (52.6)	1,313 (74.0)	461 (26.0)
July - September	460 (31.9)	982 (68.1)	645 (44.7)	797 (55.3)	1,098 (76.1)	344 (23.9)	645 (36.9)	1,104 (63.1)	881 (50.4)	868 (49.6)	1,241 (71.0)	508 (29.0)
October - December	332 (26.4)	926 (73.6)	392 (31.2)	866 (68.8)	1,044 (83.0)	214 (17.0)	311 (35.1)	576 (64.9)	385 (43.4)	502 (56.6)	655 (73.8)	232 (26.2)
**Mother received tetanus**												
No	901 (43.5)	1,171 (56.5)	1,034 (49.9)	1,038 (50.1)	1,376 (66.4)	696 (33.6)	1,214 (66.7)	606 (33.3)	1,316 (72.3)	504 (27.7)	808 (44.4)	1,012 (55.6)
Yes	677 (20.0)	2,705 (80.0)	1,088 (32.2)	2,294 (67.8)	2,956 (87.4)	426 (12.6)	961 (22.6)	3,293 (77.4)	1,544 (36.3)	2,710 (63.7)	3,650 (85.8)	604 (14.2)
**Number of ANC visit**												
None	617 (59.2)	426 (40.8)	647 (62.0)	396 (38.0)	565 (54.2)	478 (45.8)	1,095 (69.4)	482 (30.6)	1,165 (73.9)	412 (26.1)	647 (41.0)	930 (59.0)
1-3	290 (35.2)	534 (64.8)	373 (45.3)	451 (54.7)	634 (76.9)	190 (23.1)	419 (41.1)	601 (58.9)	545 (53.4)	475 (46.6)	733 (71.9)	287 (28.1)
≥4	442 (17.0)	2,153 (83.0)	776 (29.9)	1,819 (70.1)	2,310 (89.0)	285 (11.0)	661 (19.0)	2,816 (81.0)	1,150 (33.1)	2,327 (66.9)	3,078 (88.5)	399 (11.5)
**Mother's age**												
15-19	98 (41.2)	140 (58.8)	129 (54.2)	109 (45.8)	169 (71.0)	69 (29.0)	188 (50.7)	183 (49.3)	248 (66.8)	123 (33.2)	220 (59.3)	151 (40.7)
20-29	679 (31.4)	1,481 (68.6)	893 (41.3)	1,267 (58.7)	1,687 (78.1)	473 (21.9)	1,111 (34.8)	2,080 (65.2)	1,490 (46.7)	1,701 (53.3)	2,370 (74.3)	821 (25.7)
30-39	449 (26.4)	1,254 (73.6)	622 (36.5)	1,081 (63.5)	1,386 (81.4)	317 (18.6)	744 (31.3)	1,636 (68.7)	991 (41.6)	1,389 (58.4)	1,836 (77.1)	544 (22.9)
40-49	152 (31.9)	324 (68.1)	191 (40.1)	285 (59.9)	365 (76.7)	111 (23.3)	209 (46.3)	242 (53.7)	253 (56.1)	198 (43.9)	289 (64.1)	162 (35.9)
**Mother's marital status**												
Never married/in union	14 (12.6)	97 (87.4)	31 (27.9)	80 (72.1)	97 (87.4)	14 (12.6)	40 (23.0)	134 (77.0)	63 (36.2)	111 (63.8)	151 (86.8)	23 (13.2)
Married	1,333 (30.7)	3,014 (69.3)	1,752 (40.3)	2,595 (59.7)	3,412 (78.5)	935 (21.5)	2,154 (35.6)	3,899 (64.4)	2,849 (47.1)	3,204 (52.9)	4,438 (73.3)	1,615 (26.7)
Formally married	37 (26.8)	101 (73.2)	59 (42.8)	79 (57.2)	111 (80.4)	27 (19.6)	58 (34.9)	108 (65.1)	70 (42.2)	96 (57.8)	126 (75.9)	40 (24.1)
**Mother's education**												
No education	1,041 (47.4)	1,155 (52.6)	1,187 (54.1)	1,009 (45.9)	1,429 (65.1)	767 (34.9)	1,639 (60.0)	1,094 (40.0)	1,826 (66.8)	907 (33.2)	1,442 (52.8)	1,291 (47.2)
Primary	239 (27.2)	640 (72.8)	378 (43.0)	501 (57.0)	724 (82.4)	155 (17.6)	261 (28.3)	662 (71.7)	417 (45.2)	506 (54.8)	748 (81.0)	175 (19.0)
Junior/secondary	277 (14.4)	1,650 (85.6)	506 (26.3)	1,421 (73.7)	1,737 (90.1)	190 (9.9)	352 (12.9)	2,385 (87.1)	739 (27.0)	1,998 (73.0)	2,525 (92.3)	212 (7.7)
Higher/tertiary	23 (5.1)	432 (94.9)	53 (11.6)	402 (88.4)	443 (97.4)	12 (2.6)	-	-	-	-	-	-
**Mother's religion**												
Islam	1,260 (38.7)	1,995 (61.3)	1,550 (47.6)	1,705 (52.4)	2,356 (72.4)	899 (27.6)	1,844 (49.9)	1,854 (50.1)	2,149 (58.1)	1,549 (41.9)	2,300 (62.2)	1,398 (37.8)
Christian/traditionalist/others	321 (14.6)	1,883 (85.4)	575 (26.1)	1,629 (73.9)	1,978 (89.7)	226 (10.3)	408 (15.1)	2,287 (84.9)	833 (30.9)	1,862 (69.1)	2,415 (89.6)	280 (10.4)
**Access to media**												
No	1,358 (31.7)	2,923 (68.3)	1,797 (42.0)	2,484 (58.0)	3,325 (77.7)	956 (22.3)	1,748 (44.6)	2,174 (55.4)	2,177 (55.5)	1,745 (44.5)	2,574 (65.6)	1,348 (34.4)
Yes	20 (6.9)	268 (93.1)	37 (12.8)	251 (87.2)	274 (95.1)	14 (4.9)	504 (20.4)	1,967 (79.6)	805 (32.6)	1,666 (67.4)	2,141 (86.6)	330 (13.4)
**Mother use phone/internet**												
No	933 (41.1)	1,336 (58.9)	1,119 (49.3)	1,150 (50.7)	1,594 (70.3)	675 (29.7)	1,645 (51.3)	1,564 (48.7)	1,949 (60.7)	1,260 (39.3)	1,935 (60.3)	1,274 (39.7)
Yes	439 (19.1)	1,856 (80.9)	707 (30.8)	1,588 (69.2)	2,006 (87.4)	289 (12.6)	607 (19.1)	2,577 (80.9)	1,033 (32.4)	2,151 (67.6)	2,780 (87.3)	404 (12.7)
**Land ownership**												
No	339 (21.1)	1,270 (78.9)	526 (32.7)	1,083 (67.3)	1,358 (84.4)	251 (15.6)	2,036 (36.8)	3,492 (63.2)	2,654 (48.0)	2,874 (52.0)	4,006 (72.5)	1,522 (27.5)
Yes	1,239 (32.2)	2,606 (67.8)	1,596 (41.5)	2,249 (58.5)	2,973 (77.3)	872 (22.7)	216 (25.0)	649 (75.0)	328 (37.9)	537 (62.1)	709 (82.0)	156 (18.0)
**Mother had health insurance**												
No	1,562 (29.4)	3,750 (70.6)	2,093 (39.4)	3,219 (60.6)	4,199 (79.0)	1,113 (21.0)	2,232 (35.7)	4,024 (64.3)	2,952 (47.2)	3,304 (52.8)	4,593 (73.4)	1,663 (26.6)
Yes	12 (9.2)	119 (90.8)	23 (17.6)	108 (82.4)	124 (94.7)	7 (5.3)	20 (14.6)	117 (85.4)	30 (21.9)	107 (78.1)	122 (89.1)	15 (10.9)

**Ethnicity**												
**Hausa**	989 (46.4)	1,142 (53.6)	1,125 (52.8)	1,006 (47.2)	1,411 (66.2)	720 (33.8)	1,423 (57.6)	1,047 (42.4)	1,590 (64.4)	880 (35.6)	1,363 (55.2)	1,107 (44.8)
**Yoruba**	103 (17.4)	489 (82.6)	161 (27.2)	431 (72.8)	519 (87.7)	73 (12.3)	90 (14.0)	553 (86.0)	182 (28.3)	461 (71.7)	585 (91.0)	58 (9.0)
**Igbo**	48 (8.4)	525 (91.6)	111 (19.4)	462 (80.6)	543 (94.8)	30 (5.2)	69 (7.4)	869 (92.6)	228 (24.3)	710 (75.7)	891 (95.0)	47 (5.0)
**others (Kanuri, Tiv, etc)**	441 (20.4)	1,722 (79.6)	728 (33.7)	1,435 (66.3)	1,861 (86.0)	302 (14.0)	670 (28.6)	1,672 (71.4)	982 (41.9)	1,360 (58.1)	1,876 (80.1)	466 (19.9)
**Sex of household head**												
**Female**	73 (19.9)	294 (80.1)	128 (34.9)	239 (65.1)	316 (86.1)	51 (13.9)	141 (21.0)	531 (79.0)	245 (36.5)	427 (63.5)	572 (85.1)	100 (14.9)
**Male**	1,508 (29.6)	3,584 (70.4)	1,997 (39.2)	3,095 (60.8)	4,018 (78.9)	1,074 (21.1)	2,111 (36.9)	3,610 (63.1)	2,737 (47.8)	2,984 (52.2)	4,143 (72.4)	1,578 (27.6)
**Household wealth**												
**Poorest/poor**	1,126 (39.9)	1,696 (60.1)	1,362 (48.3)	1,460 (51.7)	1,994 (70.7)	828 (29.3)	1,553 (53.5)	1,348 (46.5)	1,830 (63.1)	1,071 (36.9)	1,686 (58.1)	1,215 (41.9)
**Middle**	265 (23.0)	886 (77.0)	425 (36.9)	726 (63.1)	970 (84.3)	181 (15.7)	395 (29.8)	929 (70.2)	579 (43.7)	745 (56.3)	1,057 (79.8)	267 (20.2)
**Rich/richest**	190 (12.8)	1,296 (87.2)	338 (22.7)	1,148 (77.3)	1,370 (92.2)	116 (7.8)	304 (14.0)	1,864 (86.0)	573 (26.4)	1,595 (73.6)	1,972 (91.0)	196 (9.0)
**Access to bank account**												
**No**	1,282 (36.4)	2,238 (63.6)	1,627 (46.2)	1,893 (53.8)	2,613 (74.2)	907 (25.8)	2,182 (41.3)	3,103 (58.7)	2,792 (52.8)	2,493 (47.2)	3,643 (68.9)	1,642 (31.1)
**Yes**	96 (9.1)	961 (90.9)	208 (19.7)	849 (80.3)	994 (94.0)	63 (6.0)	70 (6.3)	1,038 (93.7)	190 (17.1)	918 (82.9)	1,072 (96.8)	36 (3.2)
**Household size**												
**≥9 (large)**	592 (38.4)	951 (61.6)	708 (45.9)	835 (54.1)	1,123 (72.8)	420 (27.2)	530 (28.2)	1,352 (71.8)	777 (41.3)	1,105 (58.7)	1,487 (79.0)	395 (21.0)
**5-8 (medium)**	689 (26.9)	1,872 (73.1)	968 (37.8)	1,593 (62.2)	2,068 (80.7)	493 (19.3)	966 (32.8)	1,978 (67.2)	1,325 (45.0)	1,619 (55.0)	2,234 (75.9)	710 (24.1)
**≤4 (small)**	300 (22.1)	1,055 (77.9)	449 (33.1)	906 (66.9)	1,143 (84.4)	212 (15.6)	756 (48.2)	811 (51.8)	880 (56.2)	687 (43.8)	994 (63.4)	573 (36.6)
**Length of stay in household**												
**<1/visitor**	27 (17.2)	130 (82.8)	40 (25.5)	117 (74.5)	136 (86.6)	21 (13.4)	30 (16.0)	157 (84.0)	67 (35.8)	120 (64.2)	163 (87.2)	24 (12.8)
**1-3 year**	112 (19.9)	452 (80.1)	182 (32.3)	382 (67.7)	478 (84.8)	86 (15.2)	187 (24.6)	574 (75.4)	276 (36.3)	485 (63.7)	630 (82.8)	131 (17.2)
**4-5 years**	77 (20.0)	308 (80.0)	132 (34.3)	253 (65.7)	327 (84.9)	58 (15.1)	130 (27.7)	340 (72.3)	191 (40.6)	279 (59.4)	372 (79.1)	98 (20.9)
**>5 years/always**	1,155 (33.5)	2,296 (66.5)	1,474 (42.7)	1,977 (57.3)	2,650 (76.8)	801 (23.2)	1,905 (38.3)	3,070 (61.7)	2,448 (49.2)	2,527 (50.8)	3,550 (71.4)	1,425 (28.6)
**Rural/urban**												
**Rural**	1,279 (33.0)	2,598 (67.0)	1,649 (42.5)	2,228 (57.5)	2,949 (76.1)	928 (23.9)	1,815 (43.6)	2,351 (56.4)	2,259 (54.2)	1,907 (45.8)	2,797 (67.1)	1,369 (32.9)
**Urban**	302 (19.1)	1,280 (80.9)	476 (30.1)	1,106 (69.9)	1,385 (87.5)	197 (12.5)	437 (19.6)	1,790 (80.4)	723 (32.5)	1,504 (67.5)	1,918 (86.1)	309 (13.9)
**Region of residence**												
**North west**	634 (44.6)	787 (55.4)	738 (51.9)	683 (48.1)	965 (67.9)	456 (32.1)	1,000 (56.6)	766 (43.4)	1,088 (61.6)	678 (38.4)	1,020 (57.8)	746 (42.2)
**North east**	443 (35.3)	813 (64.7)	581 (46.3)	675 (53.7)	934 (74.4)	322 (25.6)	659 (47.8)	720 (52.2)	809 (58.7)	570 (41.3)	854 (61.9)	525 (38.1)
**North central**	264 (23.5)	859 (76.5)	373 (33.2)	750 (66.8)	953 (84.9)	170 (15.1)	301 (27.2)	805 (72.8)	472 (42.7)	634 (57.3)	896 (81.0)	210 (19.0)
**South east**	39 (8.6)	417 (91.4)	96 (21.1)	360 (78.9)	432 (94.7)	24 (5.3)	65 (8.4)	705 (91.6)	208 (27.0)	562 (73.0)	726 (94.3)	44 (5.7)
**South south**	89 (15.3)	491 (84.7)	158 (27.2)	422 (72.8)	511 (88.1)	69 (11.9)	130 (18.9)	559 (81.1)	215 (31.2)	474 (68.8)	598 (86.8)	91 (13.2)
**South west**	112 (18.0)	511 (82.0)	179 (28.7)	444 (71.3)	539 (86.5)	84 (13.5)	97 (14.2)	586 (85.8)	190 (27.8)	493 (72.2)	621 (90.9)	62 (9.1)
**Livestock density index**												
**Higher**	413 (24.4)	1,279 (75.6)	597 (35.3)	1,095 (64.7)	1,399 (82.7)	293 (17.3)	1,034 (41.4)	1,465 (58.6)	1,284 (51.4)	1,215 (48.6)	1,727 (69.1)	772 (30.9)
**Medium**	526 (29.8)	1,242 (70.2)	717 (40.6)	1,051 (59.4)	1,406 (79.5)	362 (20.5)	743 (34.6)	1,405 (65.4)	1,025 (47.7)	1,123 (52.3)	1,598 (74.4)	550 (25.6)
**Small**	642 (32.1)	1,357 (67.9)	811 (40.6)	1,188 (59.4)	1,529 (76.5)	470 (23.5)	475 (27.2)	1,271 (72.8)	673 (38.5)	1,073 (61.5)	1,390 (79.6)	356 (20.4)
**Travel time**												
**Higher**	799 (36.0)	1,422 (64.0)	981 (44.2)	1,240 (55.8)	1,647 (74.2)	574 (25.8)	1,297 (51.3)	1,233 (48.7)	1,523 (60.2)	1,007 (39.8)	1,518 (60.0)	1,012 (40.0)
**Medium**	558 (29.9)	1,310 (70.1)	758 (40.6)	1,110 (59.4)	1,459 (78.1)	409 (21.9)	648 (31.4)	1,416 (68.6)	917 (44.4)	1,147 (55.6)	1,606 (77.8)	458 (22.2)
**Small**	224 (16.4)	1,146 (83.6)	386 (28.2)	984 (71.8)	1,228 (89.6)	142 (10.4)	307 (17.1)	1,492 (82.9)	542 (30.1)	1,257 (69.9)	1,591 (88.4)	208 (11.6)
**Distance to Coastline**												
**Higher**	1,085 (39.7)	1,648 (60.3)	1,334 (48.8)	1,399 (51.2)	1,948 (71.3)	785 (28.7)	1,566 (53.9)	1,339 (46.1)	1,751 (60.3)	1,154 (39.7)	1,703 (58.6)	1,202 (41.4)
**Medium**	306 (19.4)	1,268 (80.6)	471 (29.9)	1,103 (70.1)	1,380 (87.7)	194 (12.3)	460 (23.3)	1,515 (76.7)	802 (40.6)	1,173 (59.4)	1,655 (83.8)	320 (16.2)
**Small**	190 (16.5)	962 (83.5)	320 (27.8)	832 (72.2)	1,006 (87.3)	146 (12.7)	226 (14.9)	1,287 (85.1)	429 (28.4)	1,084 (71.6)	1,357 (89.7)	156 (10.3)
**Distance to edge cult. Area**												
**Higher**	273 (20.4)	1,067 (79.6)	415 (31.0)	925 (69.0)	1,160 (86.6)	180 (13.4)	362 (22.0)	1,285 (78.0)	557 (33.8)	1,090 (66.2)	1,382 (83.9)	265 (16.1)
**Medium**	369 (22.8)	1,249 (77.2)	533 (32.9)	1,085 (67.1)	1,355 (83.7)	263 (16.3)	517 (26.9)	1,404 (73.1)	798 (41.5)	1,123 (58.5)	1,557 (81.1)	364 (18.9)
**Small**	939 (37.5)	1,562 (62.5)	1,177 (47.1)	1,324 (52.9)	1,819 (72.7)	682 (27.3)	1,373 (48.6)	1,452 (51.4)	1,627 (57.6)	1,198 (42.4)	1,776 (62.9)	1,049 (37.1)
**Distance to conflict area**												
**Higher**	987 (40.6)	1,444 (59.4)	1,147 (47.2)	1,284 (52.8)	1,701 (70.0)	730 (30.0)	1,032 (44.8)	1,270 (55.2)	1,257 (54.6)	1,045 (45.4)	1,524 (66.2)	778 (33.8)
**Medium**	288 (19.6)	1,181 (80.4)	460 (31.3)	1,009 (68.7)	1,266 (86.2)	203 (13.8)	681 (31.7)	1,467 (68.3)	991 (46.1)	1,157 (53.9)	1,643 (76.5)	505 (23.5)
**Small**	306 (19.6)	1,253 (80.4)	518 (33.2)	1,041 (66.8)	1,367 (87.7)	192 (12.3)	539 (27.7)	1,404 (72.3)	734 (37.8)	1,209 (62.2)	1,548 (79.7)	395 (20.3)
**Number wet days**												
**Higher**	186 (15.6)	1,007 (84.4)	309 (25.9)	884 (74.1)	1,054 (88.3)	139 (11.7)	218 (13.9)	1,353 (86.1)	461 (29.3)	1,110 (70.7)	1,417 (90.2)	154 (9.8)

**Medium**	261 (17.3)	1,248 (82.7)	436 (28.9)	1,073 (71.1)	1,343 (89.0)	166 (11.0)	439 (23.7)	1,413 (76.3)	731 (39.5)	1,121 (60.5)	1,550 (83.7)	302 (16.3)
**Small**	1,134 (41.1)	1,623 (58.9)	1,380 (50.1)	1,377 (49.9)	1,937 (70.3)	820 (29.7)	1,595 (53.7)	1,375 (46.3)	1,790 (60.3)	1,180 (39.7)	1,748 (58.9)	1,222 (41.1)
**Proximity to national borders**												
**Higher**	569 (28.4)	1,431 (71.5)	783 (39.1)	1,217 (60.9)	1,605 (80.3)	395 (19.8)	761 (34.6)	1,438 (65.4)	1,047 (47.6)	1,152 (52.4)	1,612 (73.3)	587 (26.7)
**Medium**	540 (31.5)	1,177 (68.5)	729 (42.5)	988 (57.5)	1,318 (76.8)	399 (23.2)	809 (36.0)	1,436 (64.0)	1,058 (47.1)	1,187 (52.9)	1,648 (73.4)	597 (26.6)
**Small**	472 (27.1)	1,270 (72.9)	613 (35.2)	1,129 (64.8)	1,411 (81.0)	331 (19.0)	682 (35.0)	1,267 (65.0)	877 (45.0)	1,072 (55.0)	1,455 (74.7)	494 (25.3)
**Land surface day time temperature**												
**Higher**	933 (37.5)	1,554 (62.5)	1,143 (46.0)	1,344 (54.0)	1,832 (73.7)	655 (26.3)	1,520 (52.5)	1,373 (47.5)	1,715 (59.3)	1,178 (40.7)	1,730 (59.8)	1,163 (40.2)
**Medium**	450 (25.6)	1,307 (74.4)	642 (36.5)	1,115 (63.5)	1,437 (81.8)	320 (18.2)	488 (24.5)	1,501 (75.5)	813 (40.9)	1,176 (59.1)	1,648 (82.9)	341 (17.1)
**Small**	198 (16.3)	1,017 (83.7)	340 (28.0)	875 (72.0)	1,065 (87.7)	150 (12.3)	244 (16.1)	1,267 (83.9)	454 (30.0)	1,057 (70.0)	1,337 (88.5)	174 (11.5)

Table S4: Factors associated with DTP1, MCV1 and composite coverage by region for DHS 2018 using multilevel binary logistic regression models

	DHS											
	North Central			North East			North West			South		
	Received DTP1	Received MCV1	Receipt of Zero dose	Received DTP1	Received MCV1	Receipt of Zero dose	Received DTP1	Received MCV1	Receipt of Zero dose	Received DTP1	Received MCV1	Receipt of Zero dose
Characteristi	OR (95%	OR (95%	OR (95%	OR (95%	OR (95%	OR (95%	OR (95%	OR (95%	OR (95%	OR (95%	OR (95%	OR (95%
Sex of child: female	0.86 (0.58-1.26)	1.08 (0.80-1.45)	1.32 (0.83-2.09)	1.10 (0.83-1.45)	1.11 (0.86-1.45)	0.85 (0.64-1.13)	1.15 (0.90-1.46)	1.14 (0.91-1.42)	0.91 (0.71-1.15)	0.77 (0.58-1.02)	0.84 (0.67-1.04)	1.27 (0.90-1.77)
SBA:	3.53 (2.22-5.70) *	1.27 (0.89-1.81)	0.24 (0.13-0.42) *	2.31 (1.58-3.40) *	2.15 (1.54-3.03) *	0.35 (0.23-0.53) *	1.49 (1.03-2.17) *	1.17 (0.83-1.64)	0.60 (0.39-0.91) *	2.12 (1.47-3.06) *	2.01 (1.49-2.71) *	0.50 (0.32-0.78) *
Birth quarter: Apr-Jun	0.91 (0.55-1.50)	0.96 (0.65-1.42)	0.62 (0.33-1.14)	0.88 (0.61-1.26)	0.88 (0.63-1.24)	1.20 (0.83-1.74)	0.86 (0.62-1.17)	1.04 (0.77-1.40)	1.26 (0.91-1.73)	0.75 (0.51-1.09)	0.65 (0.49-0.87) *	1.06 (0.67-1.66)
Birth quarter: Jul-	0.93 (0.57-1.52)	0.76 (0.52-1.12)	1.15 (0.64-2.05)	0.72 (0.50-1.03)	0.82 (0.58-1.15)	1.57 (1.08-2.27) *	1.03 (0.76-1.40)	1.03 (0.77-1.38)	1.14 (0.84-1.56)	0.78 (0.54-1.14)	0.71 (0.53-0.95) *	1.28 (0.83-1.99)
Birth quarter: Oct-Dec	0.48 (0.26-0.86) *	0.81 (0.51-1.29)	1.89 (0.96-3.71)	0.88 (0.55-1.39)	1.29 (0.84-1.97)	1.04 (0.64-1.67)	0.72 (0.48-1.08)	1.03 (0.70-1.50)	1.86 (1.24-2.78) *	0.81 (0.52-1.26)	1.12 (0.79-1.61)	0.84 (0.49-1.43)
Mother received tetanus	1.94 (1.02-3.68) *	1.08 (0.65-1.81)	0.36 (0.17-0.74) *	0.97 (0.61-1.52)	1.80 (1.15-2.83) *	0.96 (0.60-1.55)	1.99 (1.26-3.14) *	1.15 (0.75-1.76)	0.64 (0.40-1.01)	1.30 (0.83-2.03)	1.44 (0.98-2.12)	0.53 (0.32-0.88) *
ANC visits: 1-3	1.92 (0.92-4.05)	1.86 (1.02-3.40) *	0.39 (0.17-0.88) *	3.31 (1.97-5.60) *	1.38 (0.82-2.32)	0.33 (0.19-0.55) *	1.29 (0.77-2.15)	1.50 (0.92-2.44)	0.58 (0.35-0.96) *	0.73 (0.41-1.31)	0.88 (0.53-1.48)	1.14 (0.58-2.22)
ANC visits:	2.20 (1.10-4.43) *	2.11 (1.22-3.64) *	0.38 (0.17-0.81) *	5.84 (3.38-10.20) *	2.29 (1.35-3.90) *	0.17 (0.10-0.30) *	2.35 (1.41-3.90) *	2.63 (1.63-4.26) *	0.29 (0.18-0.48) *	1.67 (1.05-2.66) *	1.19 (0.80-1.78)	0.61 (0.35-1.05)
Mother's age: 20-29	1.27 (0.54-3.01)	1.76 (0.86-3.68)	0.49 (0.20-1.23)	0.98 (0.55-1.74)	1.66 (0.96-2.93)	0.60 (0.34-1.06)	1.16 (0.73-1.86)	1.56 (0.99-2.50)	0.91 (0.57-1.45)	1.65 (0.90-2.96)	2.02 (1.19-3.41) *	1.00 (0.50-2.08)
Mother's age:	1.32 (0.52-3.31)	2.59 (1.21-5.63) *	0.60 (0.22-1.65)	1.15 (0.61-2.15)	2.09 (1.15-3.89) *	0.54 (0.29-1.01)	1.21 (0.72-2.03)	1.76 (1.07-2.94) *	0.86 (0.51-1.43)	1.66 (0.89-3.04)	2.17 (1.26-3.72) *	0.82 (0.40-1.76)
Mother's age: 40-49	0.67 (0.20-2.19)	1.82 (0.68-4.91)	0.85 (0.24-3.06)	0.54 (0.24-1.19)	1.46 (0.68-3.13)	1.02 (0.46-2.24)	0.94 (0.50-1.77)	0.98 (0.52-1.82)	1.14 (0.61-2.12)	1.62 (0.72-3.68)	1.76 (0.89-3.51)	0.96 (0.36-2.52)
Mother's education:	1.11 (0.63-1.94)	1.33 (0.85-2.08)	1.10 (0.57-2.13)	2.25 (1.45-3.52) *	1.68 (1.11-2.53) *	0.31 (0.19-0.50) *	1.59 (1.07-2.38) *	1.33 (0.91-1.94)	0.66 (0.43-1.02)	1.09 (0.63-1.85)	2.24 (1.36-3.72) *	0.65 (0.36-1.17)
Mother's education: junior/higher	1.96 (1.11-3.48) *	1.82 (1.17-2.85) *	0.51 (0.25-1.03)	2.28 (1.38-3.81) *	1.39 (0.89-2.17)	0.45 (0.26-0.78) *	2.20 (1.42-3.43) *	1.70 (1.13-2.56) *	0.41 (0.25-0.67) *	1.79 (1.05-3.03) *	3.40 (2.10-5.54) *	0.34 (0.19-0.62) *
Religion: Christian/traditionalist/ot	2.63 (1.43-4.93) *	1.50 (0.97-2.32)	0.50 (0.25-1.02)	3.28 (1.87-5.86) *	1.80 (1.12-2.93) *	0.33 (0.18-0.60) *	3.14 (1.36-7.54) *	1.92 (0.98-3.79)	0.38 (0.15-0.87) *	1.25 (0.77-2.02)	1.04 (0.69-1.56)	0.82 (0.46-1.49)
Phone/internet: yes	1.38 (0.87-2.19)	1.34 (0.93-1.92)	0.63 (0.36-1.11)	1.15 (0.82-1.63)	1.12 (0.81-1.54)	0.82 (0.57-1.18)	1.63 (1.21-2.21) *	1.43 (1.07-1.91) *	0.70 (0.51-0.96) *	1.20 (0.87-1.65)	1.10 (0.84-1.44)	0.72 (0.50-1.04)
Land ownership:	0.87 (0.49-1.56)	1.01 (0.63-1.63)	1.04 (0.53-2.00)	0.85 (0.51-1.41)	1.25 (0.79-1.99)	1.18 (0.70-1.99)	1.20 (0.73-1.97)	1.00 (0.63-1.59)	0.73 (0.44-1.19)	1.24 (0.85-1.83)	1.23 (0.92-1.66)	0.77 (0.49-1.20)
Sex of household head: male	1.47 (0.75-2.85)	1.46 (0.86-2.46)	0.92 (0.42-2.10)	0.84 (0.39-1.78)	0.87 (0.44-1.73)	0.92 (0.43-2.05)	0.90 (0.49-1.65)	0.85 (0.49-1.47)	0.89 (0.48-1.69)	0.95 (0.66-1.34)	0.99 (0.75-1.30)	1.02 (0.68-1.56)
Wealth:	1.02 (0.59-1.77)	1.23 (0.80-1.90)	0.52 (0.27-1.00)	1.29 (0.83-2.01)	1.65 (1.10-2.47) *	0.79 (0.50-1.26)	1.07 (0.75-1.52)	1.16 (0.83-1.61)	0.80 (0.56-1.15)	1.45 (0.96-2.19)	1.10 (0.77-1.56)	0.79 (0.49-1.27)
Wealth: rich/richest	1.66 (0.79-3.56)	1.26 (0.73-2.19)	0.40 (0.15-1.01)	2.14 (1.12-4.13) *	2.17 (1.21-3.92) *	0.62 (0.31-1.23)	1.14 (0.71-1.84)	1.50 (0.97-2.32)	0.75 (0.45-1.24)	1.68 (1.09-2.58) *	1.68 (1.16-2.44) *	0.69 (0.42-1.14)
Access to bank	1.03 (0.48-2.29)	2.19 (1.30-3.74) *	1.70 (0.61-4.54)	1.40 (0.64-3.18)	1.90 (0.98-3.74)	0.62 (0.24-1.51)	1.93 (0.94-4.14)	1.04 (0.57-1.91)	0.48 (0.19-1.11)	3.40 (2.17-5.46) *	1.90 (1.43-2.54) *	0.17 (0.08-0.34) *
Household size: 5-8 (medium)	1.19 (0.74-1.91)	0.87 (0.60-1.24)	1.01 (0.57-1.78)	0.68 (0.46-1.01)	0.85 (0.59-1.23)	1.32 (0.88-1.99)	1.00 (0.71-1.41)	1.03 (0.75-1.43)	0.78 (0.55-1.11)	0.98 (0.71-1.34)	0.96 (0.75-1.24)	0.96 (0.66-1.40)
Household size: ≤4	0.78 (0.43-1.41)	0.80 (0.50-1.30)	1.29 (0.65-2.58)	1.08 (0.70-1.68)	1.03 (0.69-1.56)	0.83 (0.53-1.30)	0.97 (0.67-1.42)	1.09 (0.76-1.56)	0.90 (0.62-1.32)	1.32 (0.78-2.28)	1.14 (0.75-1.73)	0.81 (0.42-1.50)

**Rural/urban: urban**	0.46 (0.16-1.29)	0.42 (0.21-0.83) *	2.85 (0.81-10.20)	0.43 (0.15-1.23)	0.50 (0.20-1.20)	2.51 (0.88-7.23)	0.81 (0.44-1.50)	0.68 (0.41-1.14)	1.37 (0.76-2.45)	1.05 (0.71-1.56)	1.35 (0.96-1.89)	1.02 (0.64-1.63)
**Livestock density:**	1.50 (0.50-4.52)	0.89 (0.42-1.96)	1.17 (0.32-4.41)	1.50 (0.86-2.62)	1.21 (0.77-1.92)	0.74 (0.43-1.27)	1.57 (0.97-2.56)	1.29 (0.86-1.95)	0.70 (0.44-1.12)	1.28 (0.77-2.14)	1.36 (0.91-2.05)	0.51 (0.27-0.97) *
**Livestock density: lower**	0.90 (0.26-3.04)	1.08 (0.46-2.51)	1.76 (0.43-7.50)	1.80 (0.90-3.60)	2.17 (1.22-3.87) *	0.57 (0.29-1.11)	0.95 (0.33-2.74)	1.93 (0.83-4.52)	1.69 (0.60-4.74)	1.66 (0.99-2.76)	1.62 (1.07-2.46) *	0.48 (0.27-0.89) *
**Travel time:**	1.73 (0.86-3.53)	1.70 (1.06-2.74) *	0.54 (0.23-1.24)	1.53 (0.84-2.83)	1.65 (1.01-2.72) *	0.64 (0.35-1.15)	1.80 (1.18-2.76) *	1.31 (0.92-1.86)	0.58 (0.39-0.85) *	2.20 (1.40-3.46) *	1.23 (0.80-1.89)	0.40 (0.24-0.66) *
**Travel time: lower**	4.54 (1.39-15.55) *	2.39 (1.09-5.29) *	0.19 (0.04-0.81) *	1.89 (0.56-6.45)	2.38 (0.86-6.62)	0.67 (0.20-2.32)	1.77 (0.84-3.73)	1.73 (0.93-3.26)	0.54 (0.27-1.09)	2.43 (1.38-4.29) *	1.09 (0.65-1.82)	0.25 (0.12-0.48) *
**Dist. to edge cult. area:**	2.11 (0.98-4.66)	1.27 (0.71-2.22)	0.37 (0.14-0.95) *	1.10 (0.52-2.32)	1.78 (0.96-3.34)	0.57 (0.27-1.20)	1.23 (0.47-3.26)	1.23 (0.56-2.67)	1.01 (0.40-2.60)	0.79 (0.54-1.16)	0.64 (0.46-0.89) *	1.26 (0.80-1.99)
**Dist. to edge cult. area: lower**	1.85 (0.88-3.92)	1.11 (0.63-1.92)	0.50 (0.21-1.16)	1.13 (0.54-2.35)	3.13 (1.68-5.89) *	0.81 (0.40-1.67)	1.10 (0.41-2.93)	1.08 (0.49-2.39)	1.05 (0.41-2.73)	0.75 (0.46-1.22)	0.73 (0.47-1.15)	1.29 (0.73-2.28)
**Dist. to conflicts area:**	0.61 (0.24-1.49)	1.31 (0.72-2.41)	0.93 (0.33-2.61)	0.68 (0.36-1.28)	0.62 (0.36-1.05)	1.29 (0.69-2.40)	0.98 (0.60-1.60)	0.91 (0.61-1.37)	1.01 (0.63-1.60)	1.43 (0.99-2.07)	1.27 (0.92-1.76)	0.86 (0.56-1.32)
**Dist.to conflicts area: lower**	0.82 (0.32-2.11)	1.64 (0.89-3.04)	0.77 (0.26-2.19)	0.68 (0.35-1.34)	0.81 (0.46-1.42)	1.19 (0.62-2.28)	0.94 (0.52-1.70)	0.57 (0.34-0.93) *	1.28 (0.73-2.24)	1.21 (0.77-1.90)	1.64 (1.10-2.44) *	0.90 (0.52-1.53)
**Proximity to borders:**	0.82 (0.25-2.60)	0.90 (0.41-1.94)	0.90 (0.24-3.53)	1.28 (0.74-2.21)	1.31 (0.84-2.09)	0.74 (0.43-1.25)	0.92 (0.52-1.64)	0.89 (0.55-1.45)	0.88 (0.51-1.52)	1.24 (0.69-2.22)	0.92 (0.56-1.51)	0.84 (0.42-1.72)
**Proximity to borders: lower**	0.28 (0.06-1.29)	0.83 (0.29-2.31)	5.63 (1.05-32.13) *	1.45 (0.79-2.70)	1.78 (1.06-2.99) *	0.87 (0.48-1.59)	1.08 (0.60-1.97)	1.11 (0.67-1.85)	0.64 (0.36-1.13)	0.88 (0.45-1.72)	0.56 (0.32-0.97) *	1.39 (0.65-3.04)

Table S5: Factors associated with DTP1, MCV1 and composite coverage by region for the 2021 MICS-NICS using multilevel binary logistic regression models

	MICS-NICS											
	North Central			North East			North West			South		
	Received DTP1	Received MCV1	Receipt of Zero dose	Received DTP1	Received MCV1	Receipt of Zero dose	Received DTP1	Received MCV1	Receipt of Zero dose	Received DTP1	Received MCV1	Receipt of Zero dose
Characteristi	OR (95%	OR (95%	OR (95%	OR (95%	OR (95%	OR (95%	OR (95%	OR (95%	OR (95%	OR (95%	OR (95%	OR (95%
Sex of child: female	0.92 (0.67-1.28)	0.91 (0.69-1.20)	0.86 (0.59-1.27)	0.85 (0.63-1.15)	1.09 (0.83-1.43)	1.14 (0.85-1.53)	1.12 (0.86-1.45)	1.40 (1.08-1.82) *	0.72 (0.55-0.95) *	0.76 (0.56-1.04)	0.80 (0.63-1.02)	1.33 (0.94-1.89)
SBA:	1.14 (0.72-1.79)	1.00 (0.68-1.46)	0.91 (0.51-1.61)	1.22 (0.78-1.89)	1.09 (0.74-1.59)	0.55 (0.34-0.88) *	1.10 (0.75-1.63)	1.13 (0.76-1.67)	0.88 (0.57-1.34)	0.93 (0.57-1.48)	0.98 (0.68-1.41)	0.91 (0.53-1.60)
Birth quarter: Apr-Jun	0.91 (0.56-1.45)	0.84 (0.57-1.24)	0.78 (0.45-1.37)	0.71 (0.46-1.10)	0.87 (0.59-1.28)	1.02 (0.67-1.57)	1.21 (0.85-1.75)	1.06 (0.73-1.54)	0.70 (0.48-1.02)	1.03 (0.67-1.59)	1.05 (0.75-1.46)	0.98 (0.61-1.59)
Birth quarter: Jul-	0.94 (0.58-1.52)	0.81 (0.55-1.20)	1.09 (0.63-1.91)	0.64 (0.42-0.96) *	0.60 (0.42-0.88) *	1.34 (0.89-2.02)	1.14 (0.80-1.61)	0.94 (0.66-1.33)	0.86 (0.60-1.23)	0.77 (0.49-1.20)	0.74 (0.53-1.04)	1.30 (0.80-2.12)
Birth quarter: Oct-Dec	0.64 (0.39-1.02)	1.03 (0.69-1.54)	1.14 (0.66-2.00)	0.78 (0.49-1.23)	1.46 (0.97-2.19)	1.10 (0.70-1.73)	1.63 (1.11-2.40) *	2.44 (1.65-3.63) *	0.49 (0.32-0.74) *	1.10 (0.69-1.75)	1.54 (1.07-2.24) *	0.63 (0.36-1.08)
Mother received tetanus	3.35 (1.81-6.25) *	2.74 (1.58-4.81) *	0.20 (0.10-0.42) *	2.21 (1.06-4.64) *	1.71 (0.88-3.33)	0.36 (0.18-0.72) *	2.51 (1.58-4.02) *	2.43 (1.50-3.98) *	0.32 (0.20-0.52) *	6.07 (3.41-10.89) *	3.74 (2.25-6.26) *	0.21 (0.11-0.39) *
ANC visits: 1-3	0.81 (0.40-1.60)	0.81 (0.43-1.49)	1.11 (0.50-2.44)	1.15 (0.53-2.47)	1.20 (0.59-2.42)	0.77 (0.38-1.59)	0.70 (0.42-1.16)	0.72 (0.42-1.22)	1.47 (0.88-2.46)	1.18 (0.57-2.48)	0.69 (0.37-1.26)	0.83 (0.36-1.86)
ANC visits:	1.48 (0.75-2.89)	1.12 (0.61-2.02)	0.79 (0.36-1.73)	1.56 (0.72-3.38)	1.23 (0.61-2.48)	0.84 (0.40-1.77)	1.56 (0.95-2.54)	0.91 (0.54-1.52)	0.77 (0.46-1.29)	1.49 (0.85-2.56)	1.03 (0.63-1.67)	0.63 (0.35-1.15)
Mother's age: 20-29	0.35 (0.21-0.60) *	0.45 (0.28-0.72) *	1.96 (1.09-3.54) *	0.37 (0.22-0.64) *	0.55 (0.34-0.88) *	2.35 (1.48-3.76) *	1.02 (0.65-1.60)	0.96 (0.61-1.53)	1.38 (0.86-2.24)	0.28 (0.16-0.50) *	0.60 (0.37-0.96) *	2.22 (1.20-4.11) *
Mother's age:	0.54 (0.30-0.97) *	0.55 (0.33-0.90) *	1.85 (0.96-3.58)	0.37 (0.21-0.66) *	0.59 (0.36-0.98) *	1.84 (1.11-3.07) *	1.18 (0.72-1.94)	1.06 (0.64-1.76)	1.47 (0.88-2.50)	0.35 (0.19-0.63) *	0.68 (0.42-1.11)	1.83 (0.97-3.43)
Mother's age: 40-49	0.67 (0.31-1.46)	0.64 (0.34-1.23)	1.40 (0.55-3.41)	0.53 (0.25-1.11)	0.66 (0.34-1.28)	2.09 (1.08-4.04) *	1.55 (0.86-2.81)	1.91 (1.05-3.50) *	1.17 (0.63-2.18)	0.17 (0.08-0.34) *	0.55 (0.30-0.99) *	3.51 (1.68-7.28) *
Mother's education:	1.36 (0.87-2.14)	1.07 (0.72-1.57)	0.63 (0.36-1.08)	1.84 (1.11-3.09) *	1.87 (1.20-2.93) *	0.53 (0.32-0.88) *	1.26 (0.85-1.88)	1.23 (0.83-1.85)	0.75 (0.49-1.13)	1.17 (0.67-2.00)	0.54 (0.32-0.90) *	0.61 (0.34-1.10)
Mother's education: junior/higher	2.10 (1.32-3.35) *	2.00 (1.35-2.97) *	0.67 (0.39-1.17)	3.26 (1.99-5.44) *	2.10 (1.39-3.17) *	0.32 (0.18-0.55) *	1.86 (1.13-3.06) *	2.17 (1.33-3.55) *	0.66 (0.38-1.13)	1.89 (1.12-3.17) *	1.30 (0.79-2.11)	0.42 (0.24-0.74) *
Religion: Christian/traditionalist/ot	1.99 (1.31-3.03) *	1.46 (1.04-2.07) *	0.61 (0.37-1.00)	3.55 (1.82-7.12) *	3.06 (1.81-5.27) *	0.34 (0.17-0.66) *	2.16 (0.82-5.99)	1.78 (0.72-4.52)	0.22 (0.05-0.76) *	1.65 (1.06-2.55) *	1.27 (0.85-1.88)	0.95 (0.57-1.62)
Phone/internet: yes	0.62 (0.41-0.94) *	0.69 (0.48-0.98) *	1.13 (0.69-1.85)	1.67 (1.11-2.53) *	1.29 (0.90-1.85)	0.55 (0.36-0.84) *	0.86 (0.61-1.20)	0.90 (0.64-1.26)	1.20 (0.85-1.71)	1.02 (0.66-1.57)	0.84 (0.59-1.18)	1.29 (0.79-2.13)
Land ownership:	1.05 (0.65-1.69)	1.03 (0.70-1.52)	0.57 (0.32-1.01)	0.71 (0.44-1.13)	0.92 (0.62-1.36)	1.08 (0.68-1.71)	1.20 (0.79-1.80)	1.39 (0.92-2.12)	0.76 (0.50-1.17)	1.17 (0.81-1.70)	1.04 (0.78-1.39)	0.79 (0.52-1.20)
Sex of household head: male	1.07 (0.51-2.14)	1.42 (0.79-2.50)	1.05 (0.46-2.61)	0.80 (0.25-2.33)	1.09 (0.45-2.59)	1.99 (0.60-8.26)	1.98 (0.58-6.58)	2.57 (0.78-8.71)	0.59 (0.17-2.17)	1.10 (0.71-1.66)	1.24 (0.88-1.72)	1.02 (0.64-1.65)
Wealth:	2.75 (1.71-4.49) *	1.33 (0.90-1.96)	0.39 (0.22-0.68) *	1.33 (0.81-2.20)	1.28 (0.83-1.97)	0.69 (0.43-1.11)	1.18 (0.80-1.74)	1.13 (0.76-1.66)	0.87 (0.57-1.31)	1.72 (1.13-2.63) *	1.28 (0.92-1.80)	0.72 (0.45-1.15)
Wealth: rich/richest	4.72 (2.52-9.05) *	2.59 (1.57-4.33) *	0.13 (0.05-0.29) *	1.62 (0.84-3.15)	1.48 (0.85-2.57)	0.77 (0.39-1.50)	1.35 (0.76-2.41)	1.17 (0.66-2.09)	0.61 (0.32-1.14)	2.28 (1.39-3.79) *	2.23 (1.51-3.31) *	0.47 (0.26-0.81) *
Access to bank account: yes	1.75	1.46	0.63	1.32	0.93	1.18	2.91	2.42	0.37	1.47	1.36	0.75

	(0.93-3.43)	(0.91-2.36)	(0.25-1.46)	(0.70-2.54)	(0.56-1.55)	(0.58-2.33)	(1.56-5.61) *	(1.35-4.42) *	(0.18-0.73) *	(0.91-2.41)	(0.96-1.92)	(0.43-1.30)
**Household size: 5-8 (medium)**	1.19 (0.80-1.78)	1.04 (0.74-1.47)	0.72 (0.46-1.13)	1.20 (0.84-1.71)	0.78 (0.57-1.07)	0.96 (0.68-1.34)	1.00 (0.74-1.35)	0.98 (0.72-1.33)	1.20 (0.88-1.65)	0.96 (0.59-1.54)	0.96 (0.65-1.42)	1.15 (0.68-1.98)
**Household size: ≤4**	1.34 (0.82-2.18)	1.39 (0.92-2.10)	0.58 (0.33-1.02)	1.23 (0.76-2.01)	0.86 (0.56-1.31)	0.76 (0.47-1.21)	1.04 (0.69-1.56)	0.74 (0.49-1.12)	1.52 (0.99-2.35)	1.26 (0.73-2.15)	1.11 (0.72-1.70)	0.83 (0.46-1.53)
**Rural/urban: urban**	0.22 (0.11-0.47) *	0.42 (0.24-0.75) *	3.86 (1.53-9.73) *	0.97 (0.45-2.10)	1.77 (0.96-3.32)	0.73 (0.41-1.31)	0.96 (0.51-1.82)	0.69 (0.34-1.38)	1.26 (0.66-2.41)	1.77 (1.07-2.96) *	1.31 (0.90-1.91)	0.66 (0.37-1.14)
**Livestock density:**	0.79 (0.38-1.55)	0.62 (0.37-1.02)	1.98 (0.75-6.02)	0.35 (0.11-1.03)	0.64 (0.28-1.47)	3.53 (1.24-11.95) *	0.67 (0.44-1.01)	0.85 (0.49-1.46)	1.19 (0.72-1.98)	0.96 (0.63-1.47)	1.22 (0.85-1.75)	1.00 (0.61-1.61)
**Livestock density: lower**	0.38 (0.17-0.79) *	0.46 (0.26-0.82) *	4.39 (1.56-14.18) *	0.41 (0.11-1.41)	0.82 (0.31-2.11)	3.07 (0.99-11.12)	1.34 (0.77-2.31)	1.00 (0.46-2.15)	0.91 (0.44-1.90)	1.05 (0.64-1.72)	1.54 (0.97-2.44)	1.12 (0.64-1.94)
**Travel time:**	1.74 (1.16-2.64) *	1.32 (0.93-1.87)	0.79 (0.49-1.26)	0.74 (0.42-1.30)	0.63 (0.39-0.99) *	1.50 (1.01-2.23) *	0.50 (0.34-0.74) *	0.70 (0.43-1.13)	1.26 (0.80-1.98)	1.77 (1.12-2.81) *	1.66 (1.12-2.44) *	0.58 (0.35-0.97) *
**Travel time: lower**	1.61 (0.80-3.25)	1.11 (0.64-1.93)	0.56 (0.23-1.34)	0.97 (0.32-2.92)	0.77 (0.32-1.83)	1.25 (0.52-3.00)	0.77 (0.34-1.73)	1.20 (0.48-2.98)	1.11 (0.47-2.61)	1.38 (0.81-2.37)	1.82 (1.19-2.79) *	0.65 (0.35-1.19)
**Dist. to edge cult. area:**	0.91 (0.57-1.44)	0.81 (0.56-1.16)	1.43 (0.82-2.52)	1.67 (0.70-3.97)	1.51 (0.75-3.06)	0.95 (0.47-1.94)	0.67 (0.23-1.85)	1.11 (0.39-3.09)	1.57 (0.53-4.90)	1.39 (0.92-2.11)	1.07 (0.77-1.49)	0.96 (0.61-1.53)
**Dist. to edge cult. area: lower**	0.58 (0.37-0.91) *	0.71 (0.49-1.04)	1.58 (0.90-2.78)	2.17 (0.89-5.42)	1.69 (0.83-3.52)	0.72 (0.34-1.50)	1.20 (0.42-3.38)	1.33 (0.47-3.78)	1.30 (0.43-4.12)	1.10 (0.65-1.88)	0.96 (0.62-1.51)	1.34 (0.74-2.43)
**Dist. to conflicts area:**	0.99 (0.63-1.55)	0.75 (0.51-1.10)	0.78 (0.46-1.33)	1.14 (0.62-2.11)	1.11 (0.68-1.82)	0.83 (0.54-1.29)	1.36 (0.57-3.24)	1.04 (0.40-2.69)	0.85 (0.34-2.10)	1.22 (0.74-2.00)	1.32 (0.87-2.00)	0.65 (0.38-1.12)
**Dist.to conflicts area: lower**	1.56 (0.96-2.55)	0.84 (0.57-1.25)	0.32 (0.17-0.60) *	0.73 (0.36-1.45)	0.67 (0.38-1.16)	0.80 (0.47-1.40)	5.07 (1.71-15.78) *	1.97 (0.67-5.90)	0.25 (0.06-0.88) *	0.81 (0.48-1.36)	0.93 (0.58-1.47)	1.15 (0.66-2.03)
**Proximity to borders:**	1.33 (0.72-2.51)	1.28 (0.76-2.19)	1.07 (0.52-2.15)	1.30 (0.71-2.41)	1.17 (0.70-1.96)	1.21 (0.78-1.88)	1.04 (0.61-1.75)	0.93 (0.47-1.85)	0.88 (0.47-1.63)	0.56 (0.26-1.14)	1.10 (0.64-1.88)	1.51 (0.68-3.68)
**Proximity to borders: lower**	0.41 (0.21-0.82) *	0.55 (0.30-0.99) *	1.86 (0.84-4.02)	3.55 (1.60-8.06) *	2.41 (1.25-4.71) *	0.42 (0.23-0.79) *	2.26 (1.31-3.93) *	2.20 (1.09-4.48) *	0.43 (0.23-0.82) *	0.40 (0.18-0.80) *	0.69 (0.39-1.20)	1.97 (0.90-4.76)
